# Cgref1 is a CREB-H-regulated hepatokine that promotes hepatic *de novo* lipogenesis by mediating epididymal fat insulin resistance

**DOI:** 10.7150/ijbs.97008

**Published:** 2025-03-24

**Authors:** Pearl Chan, Pak-Hin Hinson Cheung, Xiao-Zhuo Kang, Yun Cheng, Chi-Ming Wong, Dong-Yan Jin, Chi-Ping Chan

**Affiliations:** 1 School of Biomedical Sciences, The University of Hong Kong, 21 Sassoon Road, Pokfulam, Hong Kong.; 2 State Key Laboratory of Liver Research, The University of Hong Kong, 21 Sassoon Road, Pokfulam, Hong Kong.; 3 Department of Health Technology and Informatics, Hong Kong Polytechnic University, 11 Yuk Choi Road, Hung Hom, Hong Kong.

**Keywords:** Cgref1, CREB-H transcription factor, hepatokine, diabetes, metabolic syndrome, metabolic dysfunction-associated steatotic liver disease (MASLD)

## Abstract

**Rationale:** Type 2 diabetes mellitus and metabolic dysfunction-associated steatotic liver disease (MASLD) are interrelated metabolic disorders that pose significant health concerns. Hepatokines and other regulatory factors implicated in these diseases are incompletely understood. Here, we report on a new hepatokine named cell growth regulator with EF-hand domain 1 (Cgref1) that modulates lipid metabolism to aggravate these conditions.

**Methods:** *Cgref1* was identified by microarray analysis of downregulated genes in liver of *Creb3l3*^-/-^ mice. *Cgref1*^-/-^ mice were subjected to transcriptomic, metabolomic and lipidomic analyses as well as metabolic assays. Gain-of-function and loss-of-function assays were performed in primary hepatocytes and cultured human and mouse cells.

**Results:** Cgref1 expression is induced by hepatic transcription factor CREB-H. Secreted Cgref1 primarily targets epididymal white adipose tissue (eWAT), where insulin signalling and glucose uptake are suppressed. *Cgref1^-/-^* mice showed lower tendencies of developing obesity, hyperglycaemia and dyslipidaemia, associated with compromised hepatic *de novo* lipogenesis. Thus, Cgref1 poses an advantage to maintain the normal functioning of vital organs by preserving glucose from being absorbed into eWAT. However, in circumstances where Cgref1 expression becomes excessive, eWAT develops insulin resistance, which in turn promotes hepatic glucose production, lipogenesis and MASLD development.

**Conclusion:** As a hepatokine that affects blood glucose levels and lipogenesis, Cgref1 is a potential target in the intervention of metabolic disorders.

## Introduction

Around 60% of patients with type 2 diabetes mellitus (T2DM) also have metabolic dysfunction-associated steatotic liver disease (MASLD) 1. This association can be explained by two mechanisms. First, hyperglycemia arises from insulin resistance. β cell dysfunction provides excessive glucose as energy substrate for hepatic de novo lipogenesis (DNL) to occur 2. Second, the diminished sensitivity to insulin favors lipolysis in peripheral tissues. This process creates free fatty acids (FFA) and increases FFA flux to the liver 3. Therefore, in the context of T2DM, elevated glucose and FFA levels could concurrently promote steatosis by contributing to the hepatic triglyceride (TG) pool. More recently, it has been identified that hepatocyte-derived exosomal microRNAs, induced by liver injury, could also aggravate MASLD 4. Nevertheless, the root causes of insulin resistance in relation to MASLD development are not fully identified. Until recent decades, the concept of inter-organ communication in hepatokine studies has enabled alternative insights on metabolic diseases 5. Generally, hepatokines are liver-made secretory proteins that have been reported to influence lipid and glucose metabolism 6. For example, fibroblast growth factor 21 (Fgf21) could lower blood glucose by promoting insulin action on skeletal and adipose tissues 7, 8. On the contrary, fetuin A and selenoprotein P might impair insulin signalling 9, 10. Over the years, a large amount of research has been performed on certain hepatokines, such as those mentioned above, due to their remarkable biological activities. However, our general understanding of hepatokines is far from adequate. A secretome profiling analysis conducted on primary human hepatocytes suggested the presence of 691 potential hepatokines 11. Similarly, in mice, up to 25% of the liver proteome has been estimated to possess secretory functions 12. These studies have crucially indicated that many candidate hepatokines and their roles in the regulation of lipid and glucose metabolism remain unexplored. Ideally, for understanding energy homeostasis and related diseases, a broader perspective of hepatokines needs to be obtained.

Transcription factor CREB-H is primarily expressed in the liver and intestines 13, 14. To become transcriptionally active, full-length CREB-H (CREB-H-FL) undergoes proteolytic cleavage and becomes truncated at the C-terminal (CREB-H-ΔTC) 15. Many transcriptional targets of CREB-H are known to have functions in lipid and glucose metabolism 16. For examples, CREB-H induces the expression of lipoprotein lipase coactivators such as Apoa4 to boost lipolysis and regulates the production of lipid droplet proteins including Fsp27β to promote lipid droplet growth 17-19. Notably, Fgf21, and the truncated form of CREB-H itself containing the C-terminal part alone, have been reported to serve as hepatokines 20, 21. In this connection, our previous studies have focused on the regulation of CREB-H activity and the characterization of downstream targets such as fasting- and CREB-H-induced protein (FACI) 22-26. Plausibly, CREB-H might upregulate additional hepatokines to exert its regulatory roles on lipid and glucose metabolism.

In this study, we identify Cgref1 as a novel hepatokine regulated by CREB-H. Cgref1, also known as Cgr11 and hydrophobestin, is a calcium-binding protein which possesses two EF-hand motifs and a signal peptide. Whereas the EF-hand motifs of Cgref1 have been found to suppress cell growth and mediate cell-to-cell adhesion 27-29, the signal peptide is suggestive of a secretory function through the ER-to-Golgi pathway 28, 30. We define CREB-H-dependent expression of Cgref1 and provide evidence that extracellular Cgref1 is largely retained in epididymal white adipose tissue (eWAT), whereby it causes insulin resistance locally and reduces glucose uptake. Thus, our findings suggest that Cgref1 retains glucose in the blood from being absorbed into eWAT. Over time, the increased blood glucose drives hepatic DNL, resulting in hepatic steatosis, greater body fat mass as well as hepatic and systemic insulin resistance. Our findings have implications in the treatment of metabolic diseases.

## Results

### Identification of *Cgref1* as a CREB-H downstream gene

**Figure 1:**
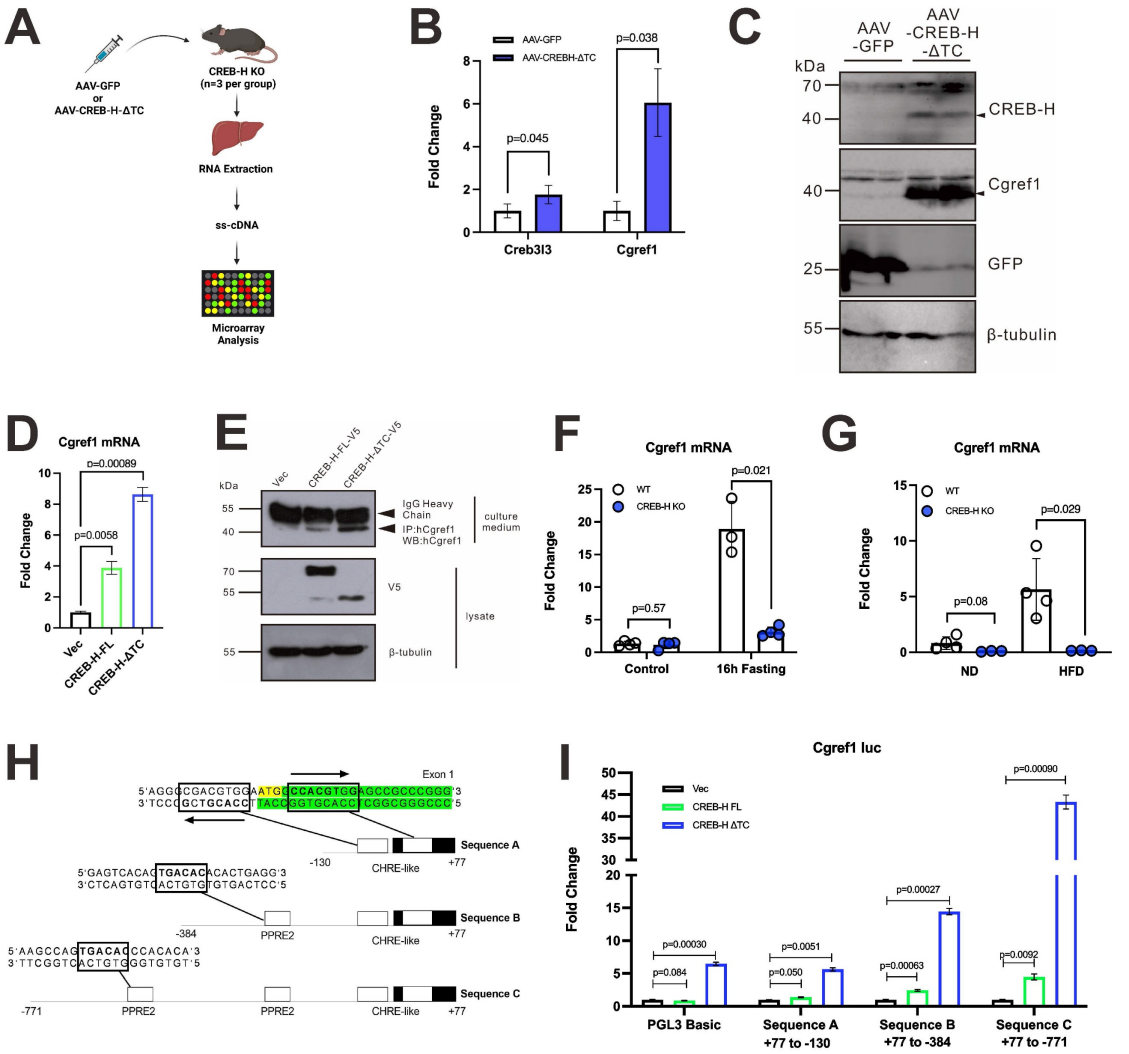
** Cgref1 expression is induced by transcription factor CREB-H.** (A) The procedure of identifying CREB-H downstream targets. AAV-CREB-H-ΔTC (or AAV-eGFP as control) was delivered to *Creb3l3*^-/-^ mice (n = 3 per group). Liver samples were processed for microarray analysis in the form of single-stranded cDNA (ss-cDNA). (B) The induction of hepatic *Cgref1* by AAV-CREB-H-ΔTC revealed by microarray analysis. (C) Western blot analysis of liver lysates overexpressed with AAV-CREB-H-ΔTC or AAV-eGFP. (D and E) RT-qPCR (D) and Western blot (E) analysis of Huh7 cell culture medium and/or lysates after transfection of CREB-H-FL-V5, CREB-H-ΔTC-V5 and vector control. (F) RT-qPCR analyses of hepatic Cgref1 expression after an overnight fast of 16 h in WT and *Creb3l3*^-/-^ mice and (G) 1 month of ND or HFD consumption in WT and *Creb3l3*^-/-^ mice. (H) The design of three luciferase reporters containing different lengths of the *Cgref1* promoter. (I) Dual-luciferase reporter assay. The three reporters harboring the *Cgref1* promoter sequence described above were co-transfected respectively with CREB-H expressing plasmids. pRL-SV40 served as internal control.

CREB-H is a transcriptional regulator of around 20 lipid and glucose metabolism genes 16-19, 32. To identify additional CREB-H-induced genes, we used liver-targeting recombinant adeno-associated virus (rAAV2/8) as a vector to deliver the CREB-H-ΔTC transgene to *Creb3l3*^-/-^ or CREB-H knockout (CREB-H KO) mice. The process of rAAV administration followed by microarray analysis was illustrated in a flow diagram (Figure 1A). Results showed that *Cgref1* transcripts in mouse livers were significantly upregulated upon CREB-H-ΔTC expression (Figure 1B). The induction of Cgref1 protein by AAV-CREB-H-ΔTC was also observed (Figure 1C). Consistently, in hepatoma cell line Huh7, the production of human homolog CGREF1 increased upon overexpression of CREB-H-FL and CREB-H-ΔTC, of which the induction by CREB-H-ΔTC was more pronounced (Figure 1D, E). In addition, CREB-H has been reported to be activated under two different physiological conditions. Firstly, by fasting and, secondly, under insulin resistant states induced by chemicals or consumption of high fat diet (HFD) 18, 32. Based on these, we recreated the same conditions using *Creb3l3*^-/-^ and wild type (WT) mice to compare hepatic Cgref1 expression. Indeed, *Cgref1* mRNA was significantly lower in *Creb3l3*^-/-^ mice that had an overnight fasting period compared to WT littermates (Figure 1F). Likewise, hepatic *Cgref1* mRNA expression in *Creb3l3*^-/-^ mice was lower whether normal diet (ND) or HFD was provided (Figure 1G). Generally consistent with previous findings 16, we observed a dose-dependent effect of free fatty acids, including palmitic acid, linoleic acid and oleic acid, on CREB-H expression, which might be the cause of Cgref1 upregulation during fasting and HFD consumption (Figure S1A).

Furthermore, we provided evidence for the correlation between CREB-H and the *Cgref1* gene from a transcriptional perspective. On the *Cgref1* promoter, two sequences, 5′-CCACGTGG-3′ and 5′-CCACGTCG-3′, which differed by the second last nucleotide and were comparable to the CREB-H-responsive element (CHRE) sequence 14, 5′- CCACGTTG-3′, were adjacent to the start codon between nucleotides -9 to +12. Additionally, two identical sequences, 5′-TGACAC-3′, encoding peroxisome proliferator-activated receptor α (PPARα)-responsive element 2 (PPRE2), to which the CREB-H-PPARα complex was reported to bind 33, were found between nucleotides -170 to -175 and -511 to -516 of the *Cgref1* gene. Thus, to see if CREB-H activates the transcriptional activity of the *Cgref1* promoter, we performed dual-luciferase reporter assay using mouse hepatoma cell line Hepa1-6. Three different lengths of the *Cgref1* promoter sequence were co-expressed with CREB-H-expressing constructs (Figure 1H, I). Strong luciferase signal was detected when exposed to CREB-H-ΔTC. These results suggested the presence of additional CREB-H interaction sites. Consistent with this, chromatin immunoprecipitation (ChIP) assay revealed that regions (-531 to -451 and -272 to -156) of the Cgref1 promoter were enriched in the DNA-protein complex containing CREB-H-ΔTC (Figure S1B). Hence, CREB-H potently induces Cgref1 expression by interacting with its promoter.

### Hepatic and serum Cgref1 are increased by HFD and secreted via ER-to-Golgi pathway

**Figure 2:**
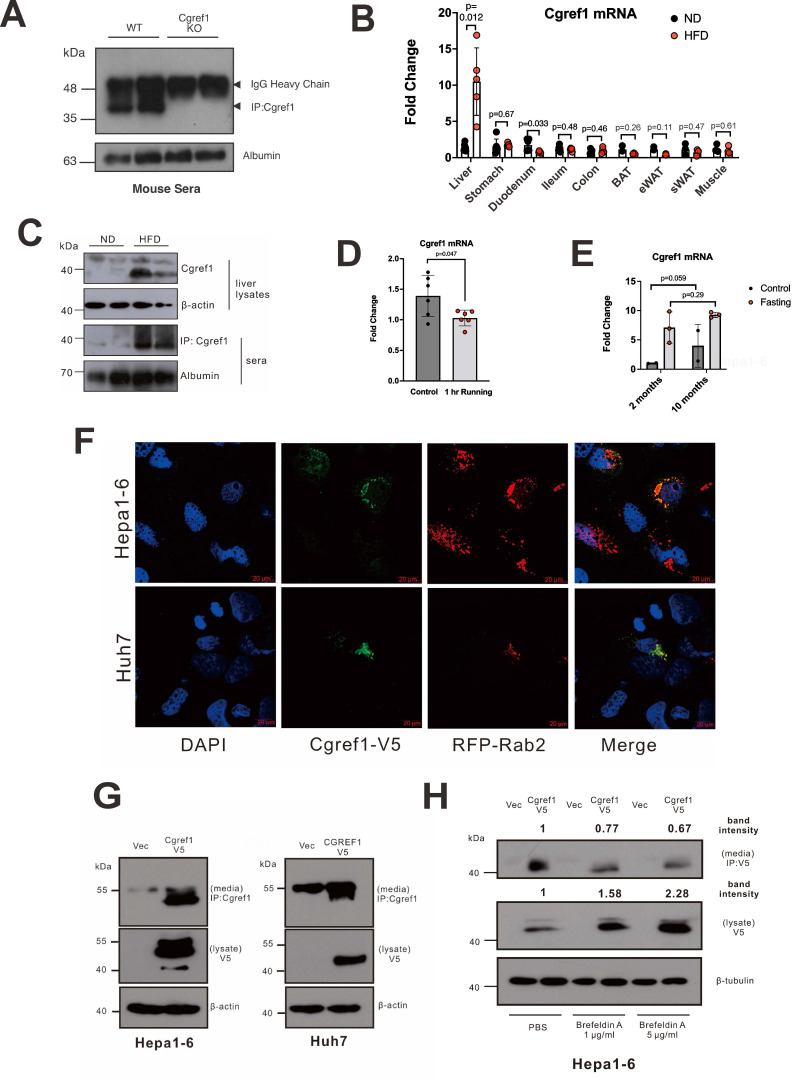
** Cgref1 expression and secretion through the ER-to-Golgi pathway.** (A) Immunoprecipitation (IP) of Cgref1 protein from mouse sera. Expression was compared by Western blot analysis. (B) RT-qPCR and (C) Western blot analyses of *Cgref1* expression in WT mouse tissues after 12 weeks of ND or HFD consumption (n = 5-6). (D) RT-qPCR analysis of hepatic *Cgref1* mRNA between resting and prolonged exercise in mice (E) and in young versus relatively aged mice. (F) Confocal microscopic analysis of RFP-Rab2 (red) and Cgref1-V5 (green) overexpression in Hepa1-6 and Huh7 cells. Scale bars at 20 μm. (G) Western blot analysis of cell culture media and lysates of Hepa1-6 and Huh7 transfected with Cgref1-V5 or CGREF1-V5. For cell culture media, IP was performed to capture V5-tagged proteins before visualizing protein expression. (H) Western blot analysis of increasing doses of brefeldin A treatment. Hepa1-6 cells were first transfected with Cgref1-V5. Two days later, different doses of brefeldin A were applied to the cells and further incubated for 6 h. Cgref1-V5 in cell culture media was immunoprecipitated. Total protein was extracted from lysates. Band intensities were analyzed by ImageJ and normalized by the values of the PBS control sample.

Having learned that Cgref1 increased endogenously in the liver upon CREB-H activation, we were interested to find out Cgref1 expression in serum and gastrointestinal tissues as CREB-H has been known to express in both liver and intestines 14, 34. Indeed, Cgref1 was present in mouse serum, which suggested protein secretion from tissues into the blood (Figure 2A). Particularly, we observed that HFD consumption exclusively induced hepatic *Cgref1* mRNA expression and that might have led to increased protein in the serum (Figure 2B, C). Notably, *Cgref1* transcription was not induced in stomach, ileum, colon, adipose tissues or skeletal muscle in HFD-fed mice (Figure 2B). In further support of this expression pattern, increased Cgref1 protein was observed in hepatic tissues subjected to immunohistochemical (IHC) staining. Cgref1 was more abundant in areas with higher density of lipids and around the portal veins, where the protein might be secreted into the circulation (Figure S2). In addition, whereas Cgref1 expression was diminished after the mice were running for one hour (Figure 2D), it was elevated in aged mice of 10 months old (Figure 2E). Cgref1 has a highly conserved signal peptide (Figure S3), consistent with secretion of the protein. To further verify the secretory function of hepatic Cgref1, several lines of experiments were performed. First, RFP-tagged ER-to-Golgi transport marker Rab2 GTPase 34 was co-expressed with Cgref1, or human homolog CGREF1, in Hepa1-6 and Huh7 cells respectively. Co-localization of RFP-Rab2 and Cgref1/CGREF1-V5 was observed in both cell lines by confocal staining (Figure 2F). Second, the transfection of CREB-H-FL, ΔTC or Cgref1/CGREF1 in hepatoma cell lines enabled the detection of Cgref1/CGREF1 protein in the cell culture media (Figure 1E and Figure 2G). Third, by treating Hepa1-6 cells with brefeldin A, a chemical known to block protein secretion by disrupting the Golgi apparatus 35, the level of extracellular Cgref1 was reduced (Figure 2H). Thus, Cgref1 was secreted through the ER-to-Golgi pathway and hepatic Cgref1 secretion was enhanced by HFD consumption.

### Cgref1 promotes obesity in mice by impairing glucose and lipid homeostasis

**Figure 3:**
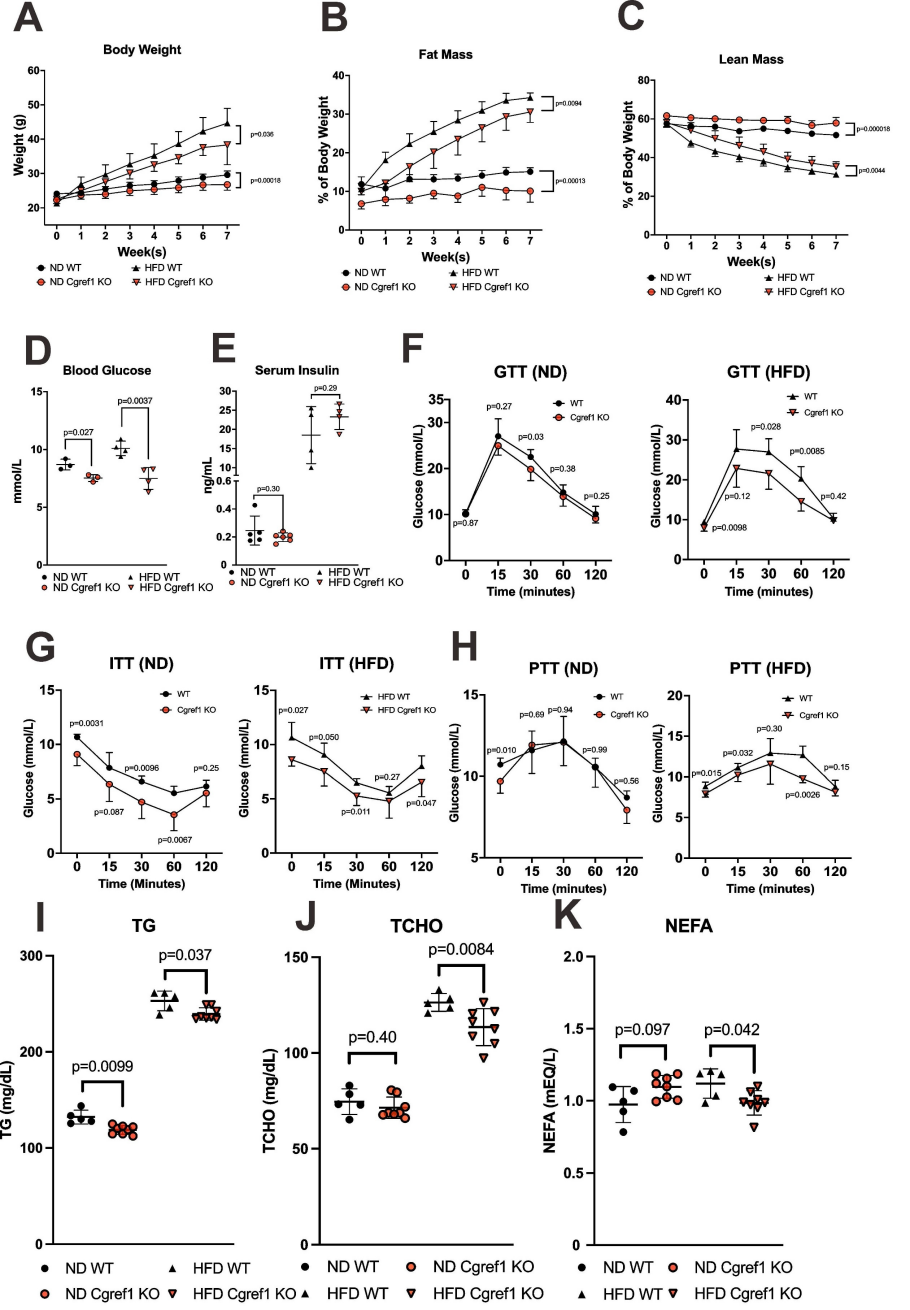
***Cgref1*^-/-^ mice exhibited a metabolically healthier phenotype.** (A) Body weights of WT and *Cgref1*^-/-^ mice at 8 weeks old on ND (n = 10-11 per group) or HFD (n = 7-8 per group) were measured on a weekly basis for 7 consecutive weeks. (B and C) Fat mass (B) and lean mass (C) of WT and *Cgref1*^-/-^ mice on ND (n = 10-11 per group) and HFD (n = 7-8 per group) were determined by body composition analysis and represented as percentages of their body weights. (D and E) Blood glucose (D) and insulin (E) levels of WT and *Cgref1*^-/-^ mice. (F-H) Intraperitoneal glucose tolerance (F), insulin tolerance (G) and pyruvate tolerance (H) tests were performed on WT and *Cgref1*^-/-^ mice on ND (n = 6-8 per group) and HFD (n = 5-8 per group). (I-K) Serum TG (I), TCHO (J) and NEFA (K) levels were measured for WT and *Cgref1*^-/-^ mice on ND (n = 5-8 per group) and HFD (n = 5-8 per group).

One of the major objectives of this study was to identify the phenotype ascribed to Cgref1. To obtain a basic understanding of Cgref1-related physiological effects, a range of metabolic tests were conducted on WT and *Cgref1^-/-^* mice. Especially, as HFD-fed WT mice had dramatically increased hepatic Cgref1 expression (Figure 1G and Figure 2B, C), we made comparisons between WT and *Cgref1^-/^*^-^ littermates after feeding ND or HFD for 7 consecutive weeks. First of all, *Cgref1^-/-^* mice were less predisposed to obesity. In terms of body weight, both HFD-fed and ND-fed *Cgref1^-/-^* mice had slower weight gain (Figure 3A). Also, regardless of the diet consumed, *Cgref1^-/-^* mice had less fat mass and more lean mass than WT controls, as revealed by body composition analysis (Figure 3B, C). Secondly, regarding glucose homeostasis, *Cgref1^-/-^* mice universally exhibited lower blood glucose levels, although insulin levels were similar to those of WT mice (Figure 3D, E). In addition, *Cgref1^-/-^* mice exhibited better glucose and insulin tolerance (Figure 3F, G). For pyruvate tolerance test, the performance of ND-fed *Cgref1^-/-^* and WT mice were similar, whereas HFD-fed *Cgref1^-/-^* mice exhibited significantly reduced gluconeogenesis compared to WT controls (Figure 3H). This result suggested that, especially under the challenge of HFD, the loss of Cgref1 somehow delayed MASLD development, which usually correlated with elevated hepatic glucose production 36. Thirdly, serum lipids were measured. Remarkably, both ND- and HFD-fed *Cgref1^-/-^* mice had lower levels of serum TG (Figure 3I), a marker known for not only overweight, but also metabolic diseases including T2DM and MASLD 36-39. Other lipids such as total cholesterol (TCHO) and non-esterified fatty acids (NEFA) or FFA levels were also lower among HFD-fed *Cgref1^-/-^* mice than WT controls (Figure 3J, K). Therefore, in short summary, an overall metabolically healthier phenotype including the reduced tendency of acquiring hyperglycemia and dyslipidemia in *Cgref1^-/-^* mice indicated that Cgref1 might be an underlying factor of metabolic disease pathogenesis.

### Cgref1 impairs insulin signalling and suppresses glucose uptake at eWAT

**Figure 4:**
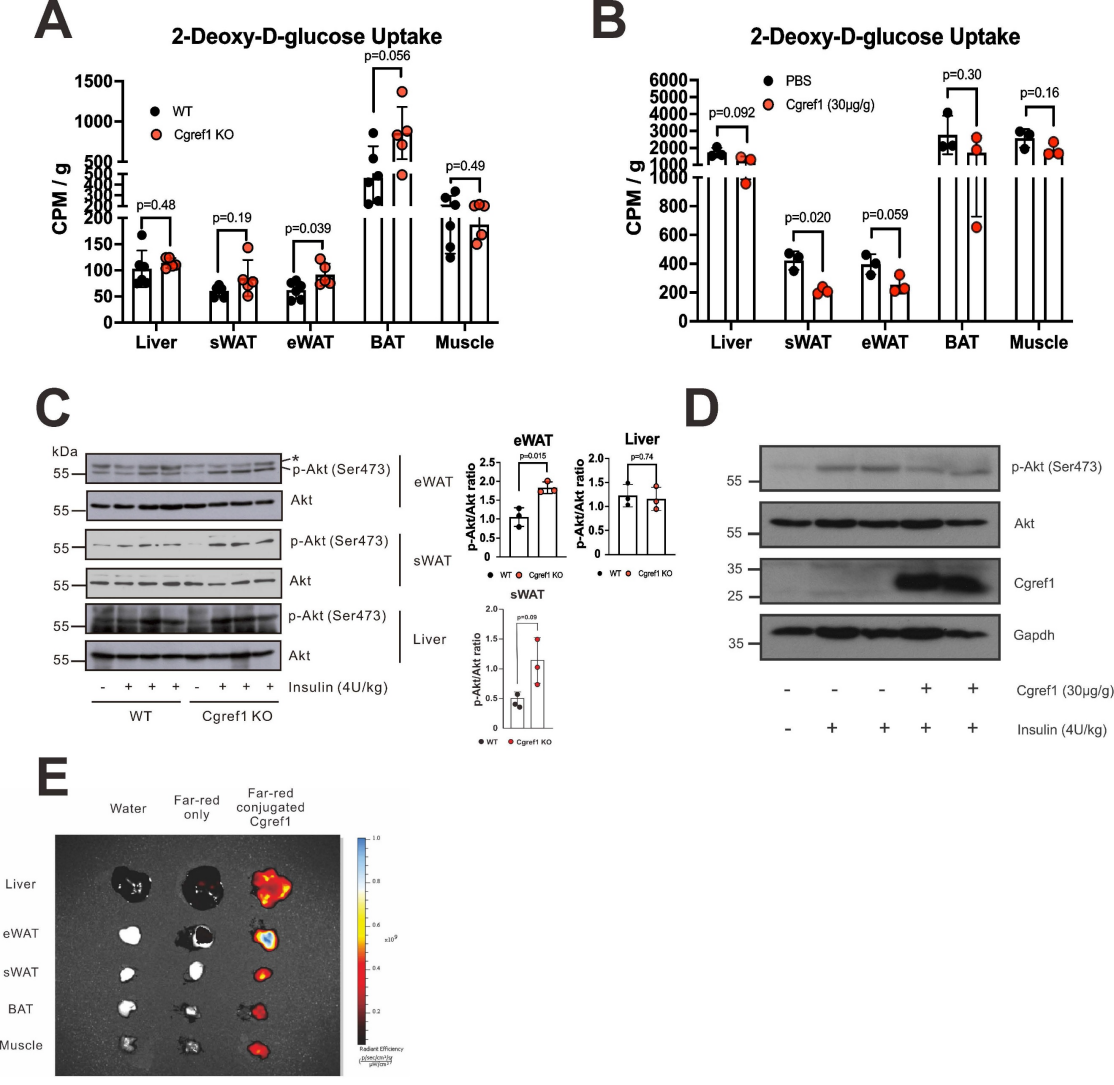
** Cgref1 targets eWAT and suppresses insulin-mediated glucose uptake.** (A) *In vivo* glucose uptake assay. Fasted WT and *Cgref1*^-/-^ mice (n = 5-6 per group) were intraperitoneally injected with ^3^H-labelled 2-DG at a dose of 20 µCi. 30 minutes later, the mice were dissected. Extracted tissues were homogenized and subjected to scintillation counting. (B) *In vivo* glucose uptake assay with exogenous Cgref1 protein supplementation. Fasted WT mice (n = 3 per group) were intraperitoneally injected with recombinant Cgref1 protein at a dose of 30 µg/g of body weight 30 minutes before the injection of 2-DG. Remaining steps were identical to those described above. (C) Western blot analysis of Akt S473 phosphorylation in eWAT, sWAT and livers of WT and *Cgref1*^-/-^ mice. Fasted mice were intraperitoneally injected with a lethal dose of insulin (4U/kg) and euthanized after 15 minutes. A non-specific band is highlighted by a star (*). (D) Western blot analysis of Akt S473 phosphorylation in eWAT of *Cgref1*^-/-^ mice. Mice were intraperitoneally injected with recombinant Cgref1 protein (30 µg/g) or PBS. Insulin (4U/kg) was injected after 20 to 30 minutes. After a further 15 minutes, mice were euthanized. (E) Recombinant Cgref1 protein conjugated to a Far Red fluorescent dye was intraperitoneally injected into a WT mouse subject. Double-distilled water or the fluorescent dye alone was injected into control mice. After 30 minutes, the animals were euthanized and their tissues were arranged for fluorescence imaging. (F) Western blot analysis of insulin signaling pathway components in eWAT of WT and Cgref1^-/-^ mice. Fasted mice were injected with insulin (4U/kg) and euthanized after 15 minutes.

High blood glucose was an indication of insulin resistance and impaired glucose uptake 40. As phenotype analysis suggested the correlation of Cgref1 with hyperglycemia and undermined glucose homeostasis (Figure 3D-H), we decided to compare insulin-mediated glucose uptake between WT and *Cgref1^-/-^* mice. An initial glucose uptake assay was performed in which mice were each injected 20 µCi of tritium (^3^H)-labelled 2-deoxy-D-glucose (2-DG) solution. The liver, eWAT, subcutaneous white adipose tissue (sWAT), brown adipose tissue (BAT) and skeletal muscle were then extracted for scintillation counting. Glucose uptake at eWAT was significantly enhanced in *Cgref1^-/-^* mice (Figure 4A). To see if opposite results could be induced by Cgref1 overexpression, a subsequent glucose uptake assay was performed in which mice were injected with recombinant Cgref1 protein before 2-DG injection. Indeed, glucose uptake at eWAT was suppressed, although only marginally significant (Figure 4B). On the other hand, it was rather unexpected to see suppressed glucose uptake at sWAT. Possible explanations with relevance to the differences among adipose tissues (ATs) and experimental design that might influence glucose uptake would be provided later in the discussion section. To verify our findings, we performed further *in vivo* experiments. Protein kinase B (Akt), an important component of the insulin signalling pathway, could mediate glucose uptake in fat cells through phosphorylation 41. Firstly, we investigated the insulin-stimulated phosphorylation of Akt at residue Ser473 (S473) in mouse eWAT, sWAT and liver. Akt S473 phosphorylation was stronger in the eWAT of *Cgref1^-/-^* mice than WT controls (Figure 4C), whereas no difference was seen in the comparison of sWAT and muscle samples. Secondly, and similarly, to see if Akt S473 phosphorylation could be reversed, recombinant Cgref1 protein was injected into mice before insulin stimulation. Results showed that Akt S473 phosphorylation in eWAT was reduced upon Cgref1 protein injection (Figure 4D). In addition, to view which tissues extracellular Cgref1 might target, fluorescent-labelled Cgref1 protein was injected into the mouse subject. As revealed by fluorescence imaging analysis, eWAT was the most strongly targeted tissue among the liver and other adipose tissues (AT) (Figure 4E). Based on the above, we believed that Cgref1 could, at least, target eWAT to suppress local insulin signalling and glucose uptake.

To verify that the systemic effects we observed *in vivo* were attributed to liver-specific Cgref1, we overexpressed Cgref1 using the liver-targeting rAAV2/8 26. AAV-Cgref1 was administered intraperitoneally into *Cgref1*^-/-^ mice and different tissues were collected for Western blot analysis. We found that Cgref1 was detected in the liver, serum and eWAT, but not skeletal muscle. The expression of Cgref1 in the eWAT was more evident as shown upon longer exposure of the blot (Figure S4A). It was noteworthy that Cgref1 expression from the AAV vector was not leaky but very specific to the liver as designed 26, since the GFP marker, which was expressed from the AAV vector, was detected only in the liver, but not eWAT or skeletal muscle (Figure S4B). These results lent further support to the notion that liver-made Cgref1 could be secreted into the circulation and reach adipose tissues such as the eWAT in an endocrine manner.

We next investigated the effect of Cgref1 on hepatic cells to determine whether it might act in an autocrine fashion to modulate *de novo* lipogenesis directly. To this end, we treated liver-derived Hepa1-6 and Huh7 cells with recombinant Cgref1 protein, followed by the measurement of oil red O staining and mRNA expression of *de novo* lipogenic genes such as *Acc1*, *Acc2* and *Scd1* (Figure S5). No significant difference in lipogenesis was observed, suggesting that Cgref1 unlikely has direct effects on hepatocytes. These results are generally in keeping with the notion that Cgref1 might function primarily in the eWAT.

**Figure 5:**
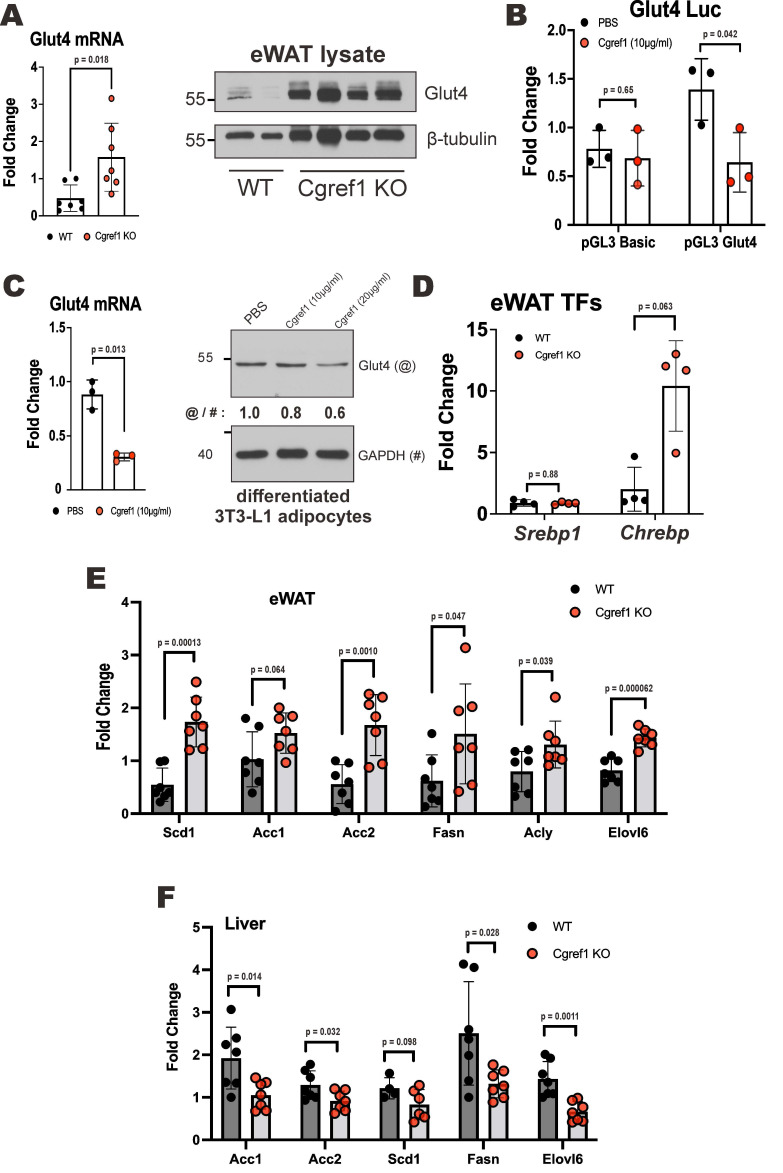
** The influence of Cgref1 on the expression of Glut4 and lipogenic factors.** (A) Glut4 mRNA and protein expression (n = 7) in eWAT of WT and *Cgref1*^-/-^ mice. (B) Dual luciferase reporter assay. Firefly luciferase reporter carrying a partial Glut4 promoter sequence was transfected into 3T3-L1 preadipocytes. A day later, the cells were incubated with Cgref1 protein (10 µg/ml) for a further 24 hours before luciferase activity measurement. pRL-SV40 served as internal control. (C) Glut4 mRNA and protein expression in differentiated 3T3-L1 adipocytes after an overnight incubation with Cgref1 protein (10 µg/ml). (D) RT-qPCR analysis of *Srebp1* and glucose-sensitive *Chrebp* mRNA in eWAT. TFs: transcription factors. (E and F) RT-qPCR analysis of lipogenic genes in eWAT (E) and liver (F).

To shed further mechanistic light on how Cgref1 affects glucose uptake, we provided evidence that Cgref1 might affect the expression of glucose transporter 4 (Glut4) at both transcriptional and protein levels. The translocation of Glut4 from the cytosol towards the cell membrane to import glucose was regulated by a signalling cascade induced by insulin 42. Depletion of Glut4 in AT could cause severe insulin resistance and increase the risk of T2DM 43. We found that *Cgref1^-/-^* mice had increased expression of Glut4 in eWAT (Figure 5A). To determine whether the difference in Glut4 expression was mainly due to the presence of Cgref1 protein but not differences in body fat mass 44, an *in vitro* dual luciferase reporter assay was performed. The experiment involved the transfection of luciferase reporter harboring a region of the Glut4 promoter sequence from -247 bp to -805 bp, which contains various binding sites of transcriptional regulators 45, in 3T3-L1 preadipocytes. The cells that were incubated with Cgref1 protein showed weakened luciferase activity (Figure 5B). Likewise, in differentiated 3T3-L1 adipocytes, the expression of Glut4 was decreased by incubation with Cgref1 protein (Figure 5C). Hence, the decreased Glut4 expression in eWAT of WT mice with normal Cgref1 expression might be partly responsible for the reduced glucose uptake. However, whether a change in Glut4 expression may lead to differences in Glut4 translocation to the cell membrane for glucose uptake requires further verification.

We went on to investigate how the lack of *Cgref1* might influence lipid homeostasis. The expression of two transcription factors in the eWAT of *Cgref1^-/-^* mice was examined. Sterol regulatory element-binding protein 1 (Srebp1) and carbohydrate response element binding protein (Chrebp) are both master regulators of lipogenic genes 46, 47. Although *Srebp1* mRNA was unaffected, *Chrebp* mRNA was boosted in the eWAT of *Cgref1*^-/-^ mice (Figure 5D). Consistently, the expression of lipogenic genes in *Cgref1^-/-^* mice was also elevated in the eWAT (Figure 5E). These genes, however, were dampened in the liver (Figure 5F). The upregulation and activation of adipose *Chrebp* occurred possibly due to increased glucose uptake 48, 49. These findings might indicate a greater glucose flux entering the eWAT of *Cgref1*^-/-^ mice which led to increased expression of Chrebp and downstream lipogenic genes. Ultimately, these changes might reduce the total amount of glucose entering the liver and subsequent hepatic lipogenesis.

### *Cgref1^-/-^* mice are less susceptible to hepatic fat accumulation

**Figure 6:**
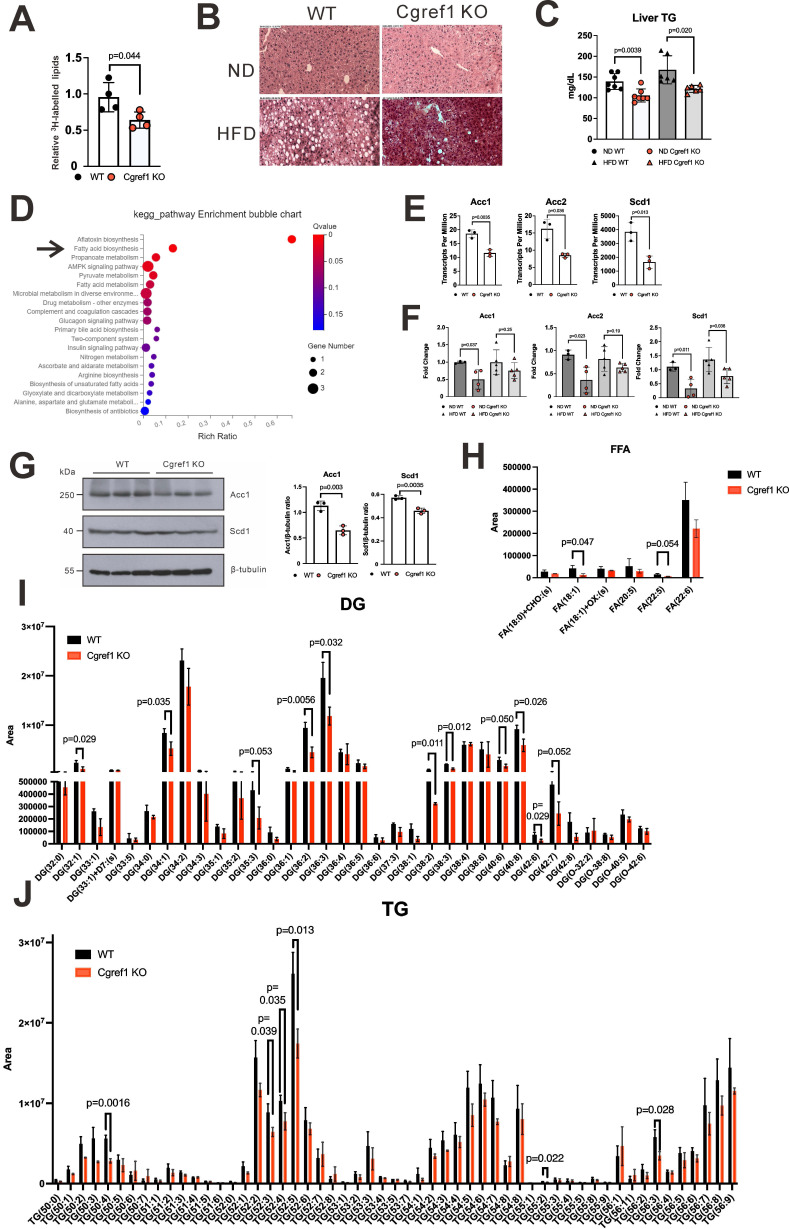
***Cgref1*^-/-^ mice are less likely to develop fatty liver.** (A) *In vivo* lipogenesis assay. Measurement of ^3^H in the lipid fraction of primary hepatocytes pre-incubated with ^3^H-labelled acetic acid (n = 4 per group). (B) H&E staining of mouse liver sections. (C) Hepatic TG measurement. (D) Total hepatic RNA of WT and *Cgref1*^-/-^ mice (n = 3 per group) were compared by RNA-seq. DEGs identified from RNA-seq were further analyzed by KEGG pathway mapping. (E) Acc1, Acc2 and Scd1 genes belong to the fatty acid biosynthesis pathway and differentially expressed between WT and *Cgref1*^-/-^ mice. (F) RT-qPCR analysis of *Acc1*, *Acc2* and *Scd1* mRNA expression in the liver cDNA of WT and *Cgref1*^-/-^ mice. (G) Western blot analysis of Acc1 and Scd1 proteins in the liver samples of WT and *Cgref1*^-/-^ mice. (H-J) Lipidomic analysis of hepatic FFA (H), DG (I) and TG (J) species identified by LC-MS/MS (n = 3 per group).

As mentioned earlier, MASLD was highly prevalent in T2DM patients 1. Having found the increased tendency of developing T2DM symptoms such as elevated blood glucose and NEFA levels in WT mice with normal Cgref1 expression, as well as other evidence showing impaired glucose homeostasis (Figure 4D-F and Figure 5A-C), it was reasonable to acknowledge that WT mice would bear higher risks of acquiring hepatic steatosis. First, we assessed the hepatic lipogenic activity of the two groups by performing an *in vivo* lipogenesis assay. In brief, primary hepatocytes were isolated from WT and *Cgref1^-/-^* mice and incubated with ^3^H-labelled acetic acid. Later, total lipids were extracted from the cells for scintillation counting. The hepatic lipids of *Cgref1^-/-^* mice contained relatively less ^3^H, which suggested a reduced lipogenic activity (Figure 6A). Furthermore, the lipid contents of mouse livers were compared. Haematoxylin and eosin (H&E) staining of liver sections showed fewer lipid deposits among HFD-fed *Cgref1^-/-^* mice (Figure 6B). Relatively, both ND- and HFD-fed *Cgref1^-/-^* mice had lower levels of hepatic TG, as revealed by colorimetric assay (Figure 6C). Consistently, RNA sequencing (RNA-seq) was performed with total hepatic RNA of WT and *Cgref1^-/-^* mice. KEGG pathway enrichment analysis of differentially expressed genes (DEGs) pointed out 'fatty acid biosynthesis' as one of the major significant pathways that differed between the test and control mice (Figure 6D). Three fatty acid biosynthesis genes — acetyl-coA carboxylase 1 (Acc1), acetyl-coA carboxylase 2 (Acc2) and stearoyl-coA desaturase 1 (Scd1) were identified as the concerning DEGs of this pathway. These genes had lower expression among *Cgref1^-/-^* mice (Figure 5F and Figure 6D, E). To validate such results, expression of the above genes was analyzed by RT-qPCR. In the ND group, *Acc1*, *Acc2* and *Scd1* mRNAs were indeed significantly lower among *Cgref1^-/-^* mice. Nevertheless, in the HFD group, although these genes also showed overall lower expression in *Cgref1*^-/-^ mice, only the difference in *Scd1* mRNA was statistically significant (Figure 6F). Correspondingly, protein expression of Acc1 and Scd1 were also weaker with reference to β-tubulin expression (Figure 6G). Last but not least, the lipid contents of the mouse livers were compared by liquid chromatography-tandem mass spectrometry (LC-MS/MS). Hepatic FFA, the product of lipolysis that associates with MASLD 50, were generally reduced (Figure 6H). In line with other observations described above, diglyceride (DG) species, the intermediate for TG synthesis, as well as TG species were generally less abundant in *Cgref1^-/-^* mice (Figure 6I, J). However, other hepatic cytosolic and membrane lipid species between WT and *Cgref1^-/-^* mice were similar (Figures S6-S8). These results indicated that *Cgref1^-/-^* mice were less inclined to accumulate hepatic fat, specifically TG and DG, but not other types of lipids.

## Discussion

In this study, we have characterized Cgref1 as a new hepatokine. In order to name a protein hepatokine, it needs to fulfil at least several criteria 6,51: 1) It is made in the liver, although not exclusively. 2) It is produced upon some kind of nutrient sensing. 3) It is a secretory protein that could circulate in the blood. 4) It exerts endocrine effects on other tissues. This action has been termed 'inter-organ crosstalk' 5. Finally, the physiological effects mediated by hepatokines are usually related to lipid and glucose metabolism 7-10, 52, 53. Relatively, here, we discuss the evidence collected for these key attributes to classify Cgref1 as a hepatokine.

Cgref1 is produced from the liver. We have demonstrated its regulation by liver-enriched transcription factor CREB-H using mouse and cell culture models (Figure 1B-I). In terms of transcriptional control, CREB-H may have more influence on the regions beyond -130bp on the *Cgref1* promoter as the luciferase signal induced by CREB-H-ΔTC in sequence A (+77bp to -130bp) was similar to that captured in the control sample in the dual-luciferase reporter assay (Figure 1I). ChIP assay also revealed regions on the *Cgref1* promoter that showed enhanced specific binding to CREB-H-ΔTC (Figure S1B). Besides and somewhat unexpectedly, we observed some expression of Cgref1 in the liver and serum of *Creb3l3^-/-^* mice (Figure 1F, G and Figure 2B). These findings suggest the possibility of other regulators so that Cgref1 can maintain its basal expression in the absence of CREB-H. It is well noted that the *Cgref1* transcript is detected in multiple tissues, especially the strong detection in gastrointestinal tissues. This has indicated that Cgref1 production is not exclusive to the liver, although its expression is induced by HFD only in the liver (Figure 2A-C). We have previously studied another CREB-H-induced gene named FACI, which also showed relatively high expression in gastrointestinal tissues 25. We believe that the expression patterns of CREB-H, FACI and Cgref1 might be correlated. For a more detailed and accurate analysis of Cgref1 in various body parts including adipose tissue, brain, skeletal muscle, brain and pancreas of mice fed with ND or HFD, Western blotting, along with ELISA and mass spectrometry might be performed to quantify actual protein levels. By enforcing liver-specific expression of Cgref1 in *Cgref1*^-/-^ mice and testing the effect of recombinant Cgref1 on cultured hepatic cells, we provided key evidence to support that hepatic Cgref1 might not have direct effects on hepatocytes and is mainly secreted into the circulation to reach the eWAT as a target tissue (Figures S4 and S5). In future studies, tissue-specific ablation of *Cgref1* mouse models may be created to assess the extent of Cgref1 secretion from each tissue type and the associated impact more precisely. As a first step, construction of liver- and intestine-specific *Cgref1*-null mice is underway.

In relation to nutrient sensing, we have shown that fasting and HFD consumption could both upregulate hepatic Cgref1 (Figure 1F, G and Figure 2B, C), as these conditions have previously been reported to stimulate CREB-H activation 18, 32. In this regard, CREB-H expression is known to be controlled by PPARα which is highly responsive to fasting and HFD consumption 55, 56. In addition, liver-specific expression of* Creb3l3* has been reported to be the lowest immediately after exercise, which then increases over a 6-hour period to stimulate gluconeogenic signalling 57. This may provide an explanation to why hepatic *Cgref1* expression falls compared to the rest group. However, whether temporarily or regularly controlling the expression of *Cgref1* through exercising could alleviate impairments in glucose and lipid homeostasis is unknown and opens up new research opportunities. Furthermore, we found that aging might positively correlate with hepatic *Cgref1* expression by comparing mice of 2 and 10 months old (Figure 2E). Rather contrastingly, a previous study reported the association of decreased liver *Creb3l3* with aging 58. Thus, further experiments are required to study Cgref1, its relationship with CREB-H and energy homeostasis in the context of aging.

The exact roles of Cgref1 in lipid and glucose metabolism remain to be defined. The lower blood glucose levels in *Cgref1*^-/-^ mice, despite similar insulin levels, might be primarily attributed to enhanced insulin signalling and glucose uptake. The absence of Cgref1 leads to AKT activation, Glu4 induction and faster glucose clearance, leading to increased insulin sensitivity. Further investigations might focus on exactly how Cgref1 suppresses Glut4 expression and insulin signalling.

Undoubtedly, Cgref1 is a secretory protein. Despite earlier publications that have demonstrated its secretory characteristic 28, 29, we have provided both *in vitro* and *in vivo* evidence to demonstrate extracellular Cgref1 expression and its secretion from hepatic tissues (Figure 1E and Figure 2A, C-H). More importantly, in this study, we have addressed the questions of where Cgref1 targets and what effects it mediates. Based on our findings, Cgref1 at least targets eWAT, where it suppresses local insulin signalling through Akt S473 phosphorylation and glucose uptake (Figure 4A-E). In support of this view, we found that Cgref1 could enter eWAT (Figure 4D, E). It was noticed that the size of Cgref1 that entered eWAT decreased compared to other detected forms (Figure 4D). We are open to the possibility that Cgref1 may undergo conformational changes under specific circumstances. Nevertheless, further studies should be conducted to address how Cgref1 interacts with adipocytes. We found in this study that Cgref1 can enter adipose tissues (Figure 4D, E). Next, we will determine whether the entry of Cgref1 is mediated by ATP-dependent processes such as receptor-binding and endocytosis, or diffusion against a concentration gradient, e.g. by using ATP synthase inhibitors such as oligomycin 59. Other methods such as subcellular fractionation, immunoprecipitation and mass spectrometry might also be used to identify the cell surface receptor for Cgref1. Furthermore, to fully understand Cgref1 effects on insulin signalling, phosphorylation of different sites of the Insr and insulin-like growth factor-1 receptor (Igf-1r) should be determined as these receptors share up to 80% homology and could form hybrid receptors 60. Pertinently, Cgref1 could reduce cell growth by suppressing mitogen-activated protein kinase activity, which is downstream of Igf-1r 29.

In terms of promoting inter-organ crosstalk, extracellular Cgref1 can control not only cell proliferation, but simultaneously the synthesis of cellular fuels by limiting glucose uptake at eWAT and possibly other ATs (Figure 4A, B). Firstly, the negative correlation between Glut4 and Cgref1 in eWAT may be due to activity or expression changes of the numerous Glut4 regulators (Figure 6A-C) 45. A wide-scope detection may be achieved by next-generation sequencing analyses. Chromatin immunoprecipitation sequencing (ChIP-seq) may reveal the binding of transcription factors on the Glut4 promoter. Alternatively, single-cell RNA-seq (scRNA-seq) that delivers results with higher definition could identify gene expression trends between the eWAT of WT and *Cgref1^-/-^* mice. Secondly, we have noticed that sWAT and BAT showed similar result patterns as eWAT to a certain degree. However, due to incompatibility of results between the two assays, we will investigate the influence of Cgref1 on these ATs separately with more appropriate experimental designs in future studies. For BAT, 18-fluorodeoxyglucose (18-FDG) is usually used for assessing glucose uptake instead of 2-DG 61. Besides, BAT is cold-sensitive and glucose uptake varies upon temperature changes 62, whereas sWAT has higher sensitivity to insulin 63. Perhaps, the sudden infusion of Cgref1 disrupted insulin signalling in sWAT to a greater extent than other tissues, which created a misleading impression of glucose uptake suppression (Figure 4B). Also, Western blot analysis of Akt S473 did not show consistency within the same genotype (Figure 4C). Thus, optimizations of Cgref1 infusion may be attempted to assess its effects in sWAT. Nonetheless, we are intrigued to see if Cgref1 may affect gene expression in different ATs relevant to adipocyte thermogenesis, an energy-expending and anti-obesity cellular mechanism 64.

We believe the development of hepatic steatosis as the secondary effect of Cgref1. With upregulated blood glucose and NEFA levels, the liver is fueled to undergo DNL (Figure 3D, K) 2, 3. To verify this phenomenon in *Cgref1^-/-^* mice, we have provided evidence that together supports a model for moderate hepatic DNL, including the downregulation of fatty acid synthesis genes (Acc1, Acc2 and Scd1) and hepatic FFA (or NEFA) as well as reduced lipogenesis activity and hepatic fat accumulation (Figure 5F and Figure 6). However, results from targeted mass spectrometry on medium and long chain fatty acids did not show significant differences between WT and *Cgref1^-/-^* mice (Figure S6). As fatty acids levels may fluctuate according to the metabolic status of the organism, further investigations on fatty acids biosynthesis are required in future studies in light of the desaturase activity of Scd1 65. In addition, no major differences were found among other hepatic lipid species between WT and *Cgref1^-/-^* mice (Figure S7 and Figure S8). This further shows the reliability of the measurement of hepatic DG and TG.

One may query, if glucose uptake decreases in the eWAT by Cgref1 protein, would lipogenesis decrease locally too? Possibly, as we have detected upregulated expression of lipogenic genes in the eWAT of *Cgref1^-/-^* mice (Figure 5E). However, the absorption of glucose into eWAT may not be completely converted into fat in the form of TG. Indeed, around 70% of the glucose absorbed is metabolized into lactate in abdominal AT, as previously reported 66. Lactate, in turn, leaves the cells as waste product, then becomes either metabolized by the liver or excreted from urine 67, 68. These may be reasons to why *Cgref1^-/-^* mice could maintain relatively less fat mass compared to WT mice despite increased glucose uptake in eWAT.

**Figure 7:**
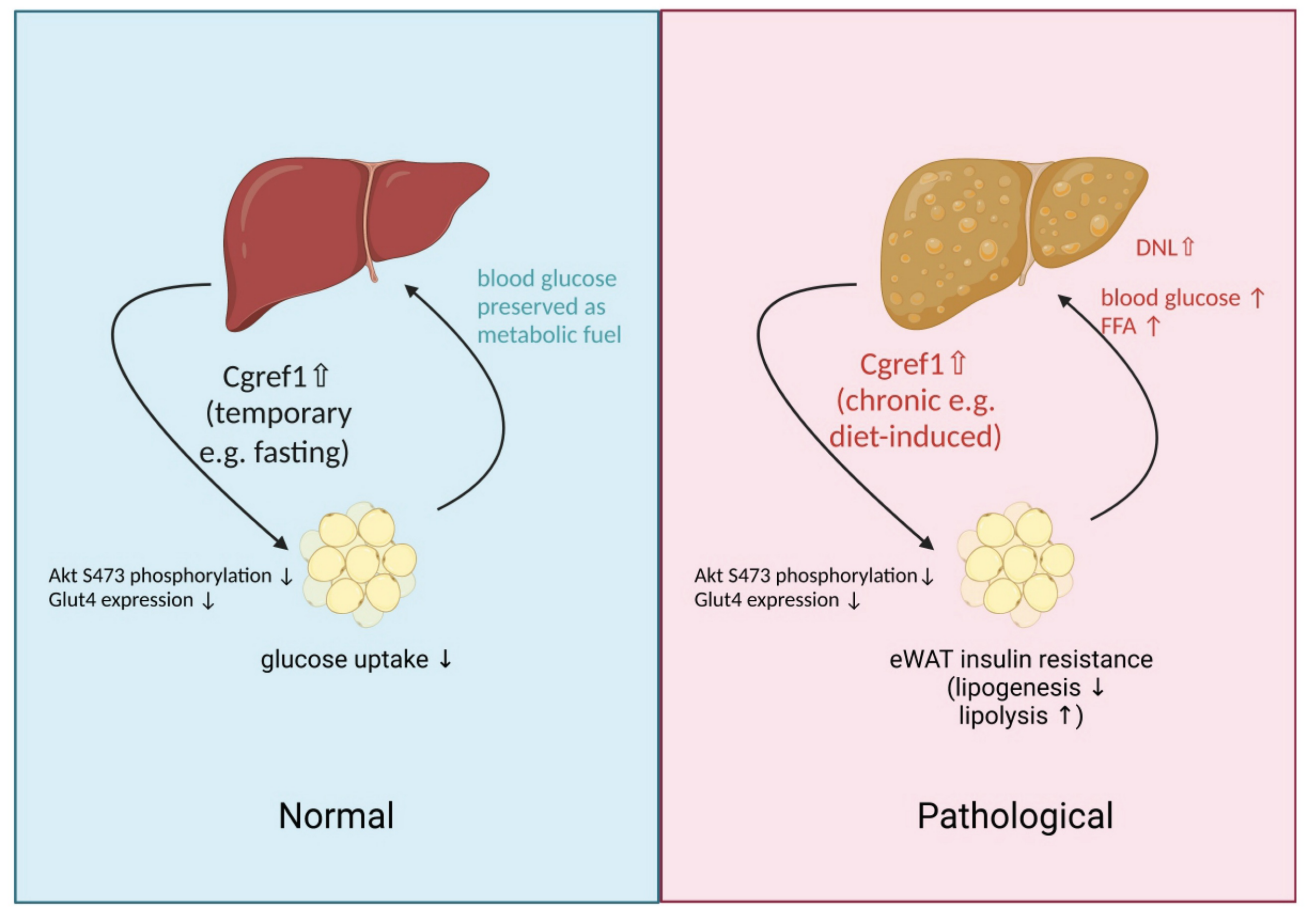
** An illustration of physiological effects mediated by Cgref1.** Liver-made Cgref1 mediates inter-organ effects of suppressing insulin signalling and glucose uptake at eWAT. Under circumstances of fasting, the secretion of Cgref1 preserves glucose from being absorbed into eWAT for the maintenance of vital organs. Under chronic and excessive expression of Cgref1, eWAT develops insulin resistance leading to elevated blood glucose and FFA levels. By processing these metabolites, hepatic DNL is upregulated and promotes hepatic fat accumulation. Simultaneously, the liver develops hepatic insulin resistance as a secondary effect. In short summary, the glucose 'turned away' from the eWAT and increased hepatic glucose production together contribute to the promotion of hyperglycemia.

Finally, it may be confusing to acknowledge that the liver produces such a protein as Cgref1 that is health deteriorating. From evolutionary and philosophical perspectives, we believe Cgref1 serves purpose in prolonging energy status that may be an advantageous effect in conditions where food supply is not guaranteed. The genetic sequences of the functional domains of *Cgref1* are highly conserved across different types of living species, which suggests similar molecular effects are mediated. By preventing the loss of glucose through absorption into eWAT and subsequent conversion into lactate, Cgref1 enables vital organs including the brain, liver and skeletal muscle to receive adequate glucose, a primary cellular fuel, for normal functioning during fasting periods e.g. due to food shortage, etc. Nevertheless, in the modern society, where lifestyle is sedentary and finding food is convenient for many, Cgref1 could be the culprit of metabolic problems such as T2DM and MASLD. An illustration of Cgref1 functions is available in Figure 7.

The systemic effects of Cgref1 may be explored in future studies. A general approach would be identifying the differentially expressed genes in specific tissues of WT and Cgref1-KO mice by transcriptome sequencing. Primary cells of various tissues may also be isolated from mice for functional analyses. For example, the isolation of primary pancreatic cells may enable the comparison of insulin and glucagon release with or without Cgref1 treatment. In addition, as we have reported that Cgref1 could target white adipose tissue, it would be interesting to find out if Cgref1 would affect its endocrine effects such as the production of leptin. Exploring receptor(s) for Cgref1 might also help to identify tissue and functional specificity of Cgref1 in regulating lipid and glucose metabolism. The current study has raised research questions that may be further investigated.

### Limitations of study

As a target of CREB-H, Cgref1 might mediate some of the known biological effects ascribed to CREB-H. In particular, it could contribute to the stimulation of DNL as previously reported 18. Astonishingly, our finding that Cgref1 may be linked to the development of MASLD is contrasting to previous reports on the protective effects of CREB-H 69-72. The deleterious effects due to excessive expression of Cgref1 remind us of the reported double-edge sword nature of CREB-H in MASLD development 19, 73. CREB-H is also known to counteract SREBP in the regulation of DNL 71. It will be of great interest to see how Cgref1 might cooperate with other CREB-H targets and metabolic regulators such as SREBP, PPARα and Fgf21 to regulate lipid and glucose homeostasis *in vivo*. In this regard, CREB-H might indeed interact with PPARα to bind with PPREs in the promoter of *Cgref1* to induce its expression (Figure 1), as in the case of other genes including FGF21, ATF4 and ATF5 33, 74.

Although its expression was robustly induced only in the liver, Cgref1 apparently maintains a basal expression in the intestine (Figure 2). The intestine is also important in lipid absorption and metabolism. The intestinal function of CREB-H and its targets such as FACI has just emerged 23, 75. It will be intriguing to determine how much Cgref1 in the blood might be secreted from the intestine and how intestinal Cgref1 might regulate DNL or serve other regulatory functions in lipid homeostasis. Although intestinal expression of Cgref1 was not induced by HFD (Figure 2), whether it might be regulated through other mechanisms merits further analysis. Plausibly, intestinal Cgref1 overexpression and intestine-specific knockout of Cgref1 might provide definitive answers to some of these important questions.

## Material and methods

### Animals

Only male mice, of 8 weeks or older, were used in this study. C57BL/6N WT mice were obtained from the Centre for Comparative Medicine Research at the University of Hong Kong. Mice were randomly assigned to treatment or control groups for all experiments without bias to any variables. Blinding was applied where appropriate. Cryopreserved sperm for producing *Cgref1*^-/-^ mice of C57BL/6NJCgref1em1(IMPC)J/Mmjax strain was purchased from the Jackson Laboratory (Bar Harbor, ME, USA). Exon 2 of the *Cgref1* gene was deleted using CRISPR Cas9 technology, which caused in a frameshift in the remaining sequence. For *Creb3l3*^-/-^ mice, cryopreserved sperm of Creb3l3tm1.1Sad/J strain was also purchased from the Jackson Laboratory (Bar Harbor, ME, USA). Exons 4 to 11 of the CREB-H gene were removed by Cre-lox recombination. Mice were housed with a daylight cycle from 7 am to 7 pm. Normal diet (ND) or high fat diet (HFD) with 60 kcal% fat (Research Diets, New Brunswick, NJ, USA) was given. Mice were analyzed on the Minispec LF90 Body Composition Analyzer (Bruker, Billerica, MA, USA) for body composition. Fasting of 6 to 12 hours was required prior to metabolic tolerance tests or the measurement of hepatic *Cgref1* mRNA transcripts. The sample size of control and test groups in this study were calculated at 90% power and at p < 0.05 between each group. In addition, no criteria were set for including and excluding animals. All animal experiments were approved by the Department of Health at the Hong Kong Special Administrative Region (License Number 20-1449 and 21-1144), and the Committee on the Use of Live Animals in Teaching and Research at the University of Hong Kong (Reference Number 4776-18 and 5258-21).

### Plasmids, key reagents and resources

Plasmids, key reagents and resources are listed in Table S1.

### Cell lines and primary cultures

Primary hepatocytes, HEK293T, 3T3-L1, Hepa1-6 and Huh-7 cell lines were cultured in Dulbecco's Modified Eagle's Medium with 10% fetal bovine serum and 1% penicillin streptomycin. All cells were maintained at 37˚C in cell culture incubators with 5% CO_2_. Plasmid transfection was performed using GeneJuice, PEI or Lipofectamine 2000 following manufacturers' protocols. Details of transfection procedures were described under individual experiments.

### Adeno-associated virus overexpression of GFP, CREB-H-ΔTC and Cgref1

The methods for producing liver-targeting recombinant adeno-associated virus 2/8 (rAAV2/8) have previously been described 26, 76. Viral capsid constructs pXX6 and p5E18-VD2/8 were cotransfected with transgene constructs pLSP1-CREB-H-ΔTC, pLSP1-Cgref1 or pLSP1-eGFP with polyethylenimine (PEI) into HEK293T cells on 15-cm culture dishes. Cloning primes were listed in Table S2. The DNA and PEI transfection ratio was 1:4. The mixture was diluted with Opti-MEM. 72 hours later, the cells were pelleted and underwent four freeze-thaw cycles. The lysate was supplemented with sodium deoxycholate (NaDOC) and benzonase followed by 2 rounds of caesium chloride (CsCl) gradient ultracentrifugation. For the first round of ultracentrifugation, 3ml of 1.5g/ml CsCl was added to the bottom of the centrifugation tube. Then, the tube was topped up with 3ml of 1.3g/ml CsCl, followed by 3ml lysate on the surface. Ultracentrifugation was performed using the P90AT rotor for 1 hour at 70,000rpm under 17^o^C. A translucent (AAV-containing) layer may be visible. For the second round of ultracentrifugation, 3ml of 1.4g/ml CsCl was added to the bottom of the tube and topped up with 6ml solution i.e. the translucent layer from the former tube. The tube was ultracentrifuged for 16 hours under 17^o^C. AAV-containing layers were concentrated using a 10K Centricon filter and PBS. qPCR was performed to estimate the virus titre. The primers (hAAT-Forward and hAAT-Reverse) are available in Table S2. 1 × 10^11^ genome copies of the virus was injected intraperitoneally into each mouse (n = 3 per group). Two weeks later, the mice were sacrificed. Liver samples were processed for further analyses.

### Chromatin immunoprecipitation (ChIP) assay

Hepa1-6 cells seeded on 150 mm plates were transiently transfected with either empty vector or 3.1A-CREB-HΔTC-V5 expression plasmid. After 40 hours, crosslinking was performed with 1% formaldehyde to stabilize DNA-protein interaction, followed by quenching and washing steps. Nuclear fraction was isolated and pelleted for chromatin. Each sample was incubated with dynabeads M280 anti-mouse magnetic beads (Invitrogen) in the presence of mouse anti-V5 antibody (ThermoFisher). Isolated chromatin was subsequently sonicated at 30% amplitude with cycles of 3 seconds on and 3 seconds off for a total of 3 minutes, before being diluted to reduce viscosity and enhance antibody binding efficiency. The product was incubated with the antibody-bound Dynabeads M280 overnight to allow specific binding of V5-tagged CREB-H-ΔTC-associated DNA fragments. Following immunoprecipitation, protein-bead complexes were washed to remove non-specific interaction. DNA was eluted from the beads and treated at elevated temperatures to reverse cross-links. Purified DNA was then subjected to qPCR to evaluate the enrichment of specific DNA regions associated with CREB-H-ΔTC. qPCR results were normalized using samples transfected with the empty vector as a negative control to account for non-specific binding. Additionally, primers targeting the coding sequence of the gene were utilized to assess non-enriched regions, ensuring that observed enrichment was specific to the promoter regions. The relative enrichment of CREB-H at promoter regions was calculated by comparing to these control samples, allowing for accurate determination of the binding of CREB-H-ΔTC with promoter region.

### DNA microarray and validation

Livers were extracted from *Creb3l3*^-/-^ mice two weeks after injecting AAV-CREB-H-ΔTC or AAV-eGFP (n=3 per group). Samples were sent to the Centre of Genomic Sciences (now called the Centre of PanorOmic Sciences/CPOS) at the University of Hong Kong for further processing and microarray analysis. The GeneChip Mouse Gene 2.0 ST Array was used. For sample processing, the GeneChip WT PLUS Reagent Kit and the GeneChip Instrument System from Applied Biosystems were used following the manufacturer's protocols. Total RNA was extracted from liver samples and converted into ss-cDNA through a series of synthetic reactions. Then, ss-cDNA was fragmented and labeled with biotin. Labelled samples were loaded onto a GeneChip Cartridge Array where ss-cDNA was hybridyzed to DNA probes. Washing and staining steps were performed using the GeneChip Fluidics Station 450. Scanning of the cartridge was performed on the GeneChip Scanner 3000. Data analysis was performed using the Transcriptome Analysis Console Software. Fold changes of genes were significantly different between the two test groups when P-value was smaller than 0.05. For validation of results, pcDNA3.1a-CREB-H-FL-V5-His, pcDNA3.1a-CREB-H-ΔTC-V5-His and pcDNA3.1a empty expression vector was transfected into Huh7 cells respectively on 6-well plates. The DNA:Genejuice ratio was 1:3. Opti-MEM was used for dilution of the transfection mixture. Two days later, RNA was extracted from the transfected cells for *Cgref1* mRNA quantification. For protein expression analysis, immunoprecipitation of Cgref1 protein was performed on the cell culture media.

### Dual-luciferase reporter assay

To see whether CREB-H has transcriptional effects on the *Cgref1* promoter, three different segments of the mouse *Cgref1* promoter sequence (-771 bp to +77 bp, -384 bp to +77 bp and -130 bp to +77 bp) were amplified from mouse genomic DNA and cloned into pGL3-Basic expression vectors via cut sites NheI and HindIII. Each of the pGL3 constructs, including the empty expression vector, was cotransfected with pRL-SV40 and either of pcDNA3.1a-V5-His empty vector, pcDNA3.1a-CREB-H-FL-V5-His or pcDNA3.1a-CREB-H-ΔTC-V5-His into Hepa1-6 cells. Plasmid DNA was transfected with Genejuice at a 1:3 ratio. The mixture was diluted using Opti-MEM. 48 hours later, the dual-luciferase reporter assay system was used to determine luciferase signals following the manufacturer's protocol.

On the other hand, a section of the Glut4 promoter sequence (-247 bp to -805 bp) of mouse origin was also cloned into pGL3-Basic expression vector via cut sites XhoI and HindIII. The cloned product or the empty expression vector, was cotransfected with pRL-SV40. After 24 hours, recombinant Cgref1 protein was added to the culture media to a final concentration of 10 µg/ml. PBS of the same volume was added to control samples. Another 24 hours later, the dual-luciferase reporter assay system was used to determine luciferase signal.

### Detection of extracellular Cgref1

Both mouse and human Cgref1 coding sequences were amplified from cDNA. The amplified fragments were cloned into pcDNA3.1c-V5-His expression vectors via cut site NotI and HindIII. pcDNA3.1c-mCgref1-V5-His (mouse) was transfected into Hepa1-6 using Genejuice. The DNA:Genejuice ratio was 1:3. pcDNA3.1c-hCGREF1-V5-His (human) was transfected into Huh7 using Lipofectamine 2000, also at a 1:3 ratio. 48 hours later, immunoprecipitation of Cgref1 protein was performed on the culture media. Protein expression in cell lysates were also analyzed. For mouse serum samples, excess IgG was removed using Recombinant Protein G Agarose before incubating with antibodies.

### Immunofluorescent staining and confocal microscopy

The processing of cells for confocal imaging has previously been described. 77. Hepa1-6 and Huh7 cells were seeded on coverslips and transfected with pAd-RFP-Rab2 and pcDNA3.1c-Cgref1-V5-His. 48 hours later, the cells were fixed with 4% paraformaldehyde and blocked with 3% bovine serum albumin (BSA) before primary antibody (anti-V5) incubation overnight. The next day, the cells were further incubated with secondary antibody (anti-mouse IgG, FITC conjugated) and DAPI. Wash steps were performed using PBS. Finally, the coverslips were mounted and visualized on the Carl Zeiss LSM 710 Confocal Microscope.

### Immunohistochemistry and H&E staining

Fixing and paraffin-embedding of mouse liver tissues have previously been described 25. Paraffin blocks were sectioned and stained in the Department of Pathology, University of Hong Kong. Immunochemical and H&E staining was performed as described 25.

### Blood biochemistry tests

Blood glucose was determined using Accu-Chek test strips and glucometer. Serum insulin was measured by the Wide Range Mouse Insulin ELISA Kit. LabAssay NEFA was used to quantify serum NEFA. Stanbio LiquiColour colorimetric assays were used to quantify serum TG and TCHO. Details of the test kits have been listed in the key resources table.

### Metabolic tolerance tests

Mice were fasted before the experiments. For glucose tolerance tests (GTT), 20% (w/v) glucose solution was prepared. The injection volume in µL was 10 times the body weight in g. For HFD groups, a 10% (w/v) glucose solution was used. In insulin tolerance tests (ITT), mice on ND were injected with an insulin dose of 0.7U/kg, whereas mice on HFD received a dose of 1.2U/kg. In pyruvate tolerance tests (PTT), all mice were injected with a sodium pyruvate dose of 2g/kg. Blood glucose was measured at several time points (0, 15, 30, 60 and 120 minutes) after injection.

### Production and purification of recombinant Cgref1 protein

Transformation of pET30a-His-Cgref1 (synthesized by Genscript) was performed using *E. coli* Rosetta-2 strain. A bacterial colony was picked and grown in Luria broth (LB) supplemented with kanamycin and chloramphenicol at 37°C with orbital shaking. When the optical density reached 0.6 to 0.8 at 600 nm, isopropyl ß-D-1-thiogalactopyranoside (IPTG) was added to a concentration of 1 mM. The culture was incubated overnight at 16°C. Bacterial cells were pelleted by centrifugation and resuspended with 10 ml NPI-10 buffer (50 mM NaH_2_PO_4_, 300 mM NaCl, 10 mM imidazole, pH = 8.0) supplemented with protease inhibitor. The solution was kept cold and sonicated at 40% amplitude for 15 minutes. The lysate was centrifuged and purified on the AKTA purifier system using a HisTrap column. Impurities were washed using NPI-20 buffer (50 mM NaH2PO4, 300 mM NaCl, 20 mM imidazole, pH = 8.0). Elution was performed using NPI-500 buffer (50 mM NaH2PO4, 300 mM NaCl, 500 mM imidazole, pH = 8.0). Cgref1 protein was concentrated using a 10k Centricon filter. The buffer was exchanged to PBS by centrifugation. The protein concentration was determined using a BSA standard curve. Proteins were ready for experiments or stored at -80°C.

### *In vivo* glucose uptake assay

Mice were fasted overnight. Each animal was intraperitoneally injected with 20 µCi of 2-deoxy-D-glucose, 2- [1,2-3H (N)] (2-DG) and euthanized after 30 minutes. The liver, sWAT, eWAT, BAT and hind leg muscle were collected with their weights recorded. The tissues were then homogenized with PBS and centrifuged at 12,000 rpm for 10 minutes. The supernatant of each sample was mixed with scintillation cocktail at 1:3.75 ratio. The mixture was subjected to tritium (^3^H) measurement by scintillation counting. For analysis, the total ^3^H count of each tissue was divided by the weight to obtain a value in ^3^H counts/gram of tissue. For measuring glucose uptake after protein injection, mice were first injected with recombinant Cgref1 protein (30µg/g of body weight). After 30 minutes, they were injected with 20 µCi of 2-DG. The remaining steps were unchanged.

### Lipogenesis assay in cultured cells

Hepa1-6 and Huh7 cells were induced by either medium containing 1% fatty acid-free BSA or 1% fatty acid-free BSA supplemented with 0.5 mM oleic acid and 0.25 mM palmitic acid for 24 hours. To assess lipogenesis, cells were fixed with 10% formalin, followed by washes and staining with oil red O. The stain was eluted with 100% isopropanol. Absorbance was measured at 500 nm on a spectrophotometer.

### *In vivo* lipogenesis assay

The procedures of mouse primary hepatocyte isolation and lipogenesis assay have previously been described 78. Primary hepatocytes were extracted and seeded at 9 × 10^4^ per well in 24-well plates. For lipogenesis assay, each well was treated with DMEM supplemented with 10% FBS, 1% penicillin streptomycin, 100 nM insulin, 10 µM acetate and 0.5µCi ^3^H-acetic acid, sodium salt, followed by a 2-hour incubation at 37^o^C in a cell culture incubator. Afterwards, cells were washed with PBS and lysed with 0.1N HCl. Total lipids were extracted from the lysate with 2:1 chloroform: methanol. Extracted lipids were mixed with 4 ml scintillation cocktail and subjected to ^3^H measurement.

### *In vivo* imaging

Two mg/ml of Cgref1 protein was labelled with the Alexa Fluor™ 647 Protein Labeling Kit according to the manufacturer's protocol. The protein was injected intraperitoneally into the mouse subject. The injection volume (µL) was 10 times the body weight of the mouse (g). On the other hand, double-distilled water and fluorescent dye were injected to the control groups respectively. After 30 minutes, the animals were sacrificed. Liver, sWAT, eWAT, BAT and hind leg muscle were collected and arranged for imaging. Images were captured on the IVIS Spectrum.

### Treadmill experiment

The details of setting up the animal treadmill and experimental conditions have been described previously 79. Briefly, mice were warmed up for 5 minutes at a speed of 5 m/min with 5° inclination before running at 14 m/min with 14° inclination for 60 minutes. Mice either ran on the treadmill or were kept in their cages and were then killed immediately.

### Protein extraction, immunoprecipitation and Western blotting

Procedures for protein expression analyses have been described previously 25, 77. For extraction, samples were lysed at 4^o^C for 20 minutes by RIPA buffer (150mM NaCl, 50mM Tris-HCl (pH = 7.4), 2mM EDTA, 1% NP-40, 0.5% NaDOC and 0.1% sodium dodecyl sulfate) supplemented with protease and phosphatase inhibitor. For animal tissues, a 1:1 RIPA (µL) : tissue (mg) ratio was required for homogenization. After centrifugation, visible fat layers were removed. Samples were normalized by the Bradford method and mixed with Protein Sample Buffer (PSB) (0.5M Tris-HCl (pH=6.8), 20% glycerol, 10% SDS and 1% saturated bromophenol blue), boiled, then analyzed or stored at -20°C. β-mercaptoethanol was supplemented to 5% of the total volume. For immunoprecipitation (IP), magnetic IgG-specific Dynabeads were pre-conjugated to primary antibodies in IP lysis buffer (150mM NaCl, 50mM Tris-HCl (pH=7.4), 2mM EDTA, 1% NP-40 and 0.5% NaDOC) at 4^o^C for 1 hour prior to sample incubation. Cell culture medium samples were centrifuged at 500 g for 2 minutes to remove cell debris and incubated overnight with the bead-antibody mix. Afterwards, samples were resuspended with PSB and boiled. Denatured protein samples were resolved through sodium dodecyl sulfate-polyacrylamide gels and immunoblotted onto polyvinylidene difluoride membranes. Blots were visualized using WesternBright ECL HRP substrate.

### RNA extraction and real-time quantitative PCR

For cell culture, 0.5 to 1 ml RNAiso Plus was added to cells and mixed thoroughly. For animal tissues, instead, 20-50 mg of tissue was homogenized with 1 ml RNAiso Plus. The remaining steps were the same and performed according to the manufacturer's protocol. Total RNA was stored at -80^o^C or reverse transcribed into cDNA using the PrimeScript RT Reagent Kit with gDNA Eraser. RT-qPCR was performed using the TB Green Premix Ex Taq II kit on the CFX96 Touch Real-Time PCR Detection System. The RT-qPCR primers used in this study are available in Table S3.

### RNA-seq and analysis

Mouse livers (n = 3 per group) were extracted with RNAiso Plus for total RNA and sent to BGI Genomics. The method of RNA-seq has previously been described 25. First, quality control tests were performed to check sample integrity. RNA concentration and quality were determined using the Qubit fluorometer, Nanodrop and the Agilent 2100 bioanalyzer before library construction. RNA sequencing was then performed using the BGISEQ-500 and the PE150 reference genome dataset. Raw data were filtered to remove low quality reads before aligning to the mouse reference genome (NCBI: GCF_000001635.26_GRCm38.p6) using HISAT 80. The average mapping ratio with reference genome was 95.54%. Next, the reads were further aligned to reference genes using Bowtie2 81. The average gene mapping ratio was 80.24%. DEGs were considered significant with a P-value under 0.05 and interpreted with pathway enrichment analysis.

### Targeted metabolomics

Mouse liver samples (n = 3 per WT and *Cgref1*^-/-^ group) were sent for analysis at the Proteomics and Metabolomics Core of the CPOS at the University of Hong Kong. For the targeted quantitation of medium and long chain fatty acids, gas chromatography-mass spectrometry (GC-MS) analysis was performed. First, a spiking solution of 100 µL chloroform and 20 µg C19:0 fatty acid internal standard was added to each sample to preserve the sample matrix. Then, the samples underwent 5 rounds of 2:1 chloroform/methanol lipid extraction, sonicated and centrifuged. The supernatant was further purified with 0.73% NaCl and methanol and dried under a nitrogen stream at 45°C. Samples were transesterified by mixing with 1 ml of methanol and 50 µl of (35%, w/w) hydrochloric acid each. Oxygen was eliminated by nitrogen overlay. Then, samples were vortexed and heated to 100°C for 1.5 h. After cooling, fatty acid methyl esters were extracted with 1 ml of hexane and 1 ml from each sample that was subjected to vortexing. 1 µl of the hexane phase was processed for GC-MS analysis 82. The Agilent 7890B GC - Agilent 7010 Triple Quadrapole Mass Spectrometer system was used. An Agilent DB-23 capillary column (60 m × 0.25 mm ID, 0.15 µm film thickness) under 33.4 psi helium pressure was used for sample separation. The GC program started at 50°C for 1 min and increased to 175°C at a ramp rate of 25°C/min. The temperature was then raised to 190°C for 5 min at a ramp rate of 3.5°C/min. The temperature was further raised to 220°C for 4 min at a ramp rate of 2°C/min. Inlet and transfer line temperatures were 250°C and 280°C respectively. Characteristic fragment ions (m/z 55, 67, 69, 74, 79, 81, 83, 87, 91, 93, 95, 96, 97, 115, 127, 143) were monitored in SIM mode. Mass spectra from m/z 50-350 were acquired in SCAN mode. The Agilent MassHunter Workstation Quantitative Analysis Software was used for data analysis. For generating linear calibration curves, the peak area ratio of external/internal standard was plotted against the standard concentration at different concentration levels. The ratio of characteristic fragment ions in the sample and standard confirmed the amount of analytes.

### Untargeted lipidomics

LC-MS/MS was performed. For sample preparation, mouse livers (n = 3 per group) were sonicated in 2 mL chloroform:methanol (2:1, v/v) on ice for 20-second 'on' and 10-second 'off' intervals. Then, samples were centrifuged at 3000 g for 5 min. The supernatants were dried under nitrogen and reconstituted with IPA:MeOH:chloroform (1:1:0.2, v/v) prior to processing on the LC-MS/MS system. A Vanquish UPLC was used for chromatographic separation. Mobile phases composed of 10 mM ammonium formate with 0.1% formic acid in acetonitrile and water, v/v 6:4, (A) and 10 mM ammonium formate with 0.1% formic acid in acetonitrile and IPA 1:9 (B) were used. A ThermoFisher Accucore C30 (2.1×150 mm, 2.6 μm) column was used. The injection volume was 3 μL and administered at a flow rate of 0.26 mL min^-1^. The temperature was 45°C. The gradient increased from 30% B to 43% B in 2 min. It was further increased to 55% B in 2.1 min, 65 % B in 12 min, 85% B in 18 min and 100 % B in 20 min. The gradient was held for 5min, then decreased linearly to 30% B for re-equilibration. The LC-MS/MS analysis was carried out on an Orbitrap Exploris 120 mass spectrometer with a HESI II probe in polar switching mode. See below for parameters: sheath gas flow rate, 60; auxiliary gas flow rate, 17; sweep gas flow rate, 1; spray voltage, +3.5/-3.0 kV; capillary temperature, 275^o^C; S-lens RF level, 70; and heater temperature, 325 °C. The dd-MS2 mode was used for data collection. Lipidsearch (Thermofisher Scientific/Mitsui Knowledge Industries) was used for data analysis using default parameters for Orbitrap MS Product Search and Alignment.

### Quantification and statistical analysis

The two-tailed student's t-test was performed to calculate the statistical significance for experiments in this study. The results presented were sample means ± SD. A p-value of 0.05 or smaller is considered statistically significant. Sample sizes have been specified for each experiment under each figure and described in the figure legends.

## Supplementary Material

Supplementary figures and tables.
