# Supplementary material for: Cgref1 is a CREB-H-regulated hepatokine that promotes hepatic de novo lipogenesis by mediating epididymal fat insulin resistance
Source: Int J Biol Sci. 2025 Mar 24;21(6):2568–88. doi: 10.7150/ijbs.97008 (PMC12035884; doi:10.7150/ijbs.97008)
Supplement: Supplementary file 1 — Supplementary figures and tables. [file ijbsv21p2568s1.pdf]

Supplementary tables and figures

Cgref1 is a CREB-H-regulated hepatokine that promotes hepatic *de novo* lipogenesis by mediating epididymal fat insulin resistance

Pearl Chan[1, 2], Pak-Hin Hinson Cheung[1,2], Xiao-Zhuo Kang[1,2], Yun Cheng[1,2], Chi-Ming Wong[3],

Dong-Yan Jin[1, 2*] and Chi-Ping Chan[1, 2*]

Supplementary tables 1-3

Supplementary figures 1-8

SUPPLEMENTAL TABLES

Table S1. Key resources table

REAGENT or RESOURCE	SOURCE	IDENTIFIER
**Antibodies**		
Rabbit Anti-Mouse Cgref1 antibody (RAISED ON REQUEST)	Genscript	N/A
Mouse Anti-Human CGREF1 (Clone 2D7)	Abnova	Cat# H00010669-M01; RRID: AB_463970
Mouse Anti-β-actin (Clone AC-74)	Sigma Aldrich	Cat# A2228; RRID: AB_476697
Mouse IgG HRP Linked Whole Ab	Cytiva	Cat# GENA931-1ML; RRID: AB_3068585
Rabbit IgG HRP Linked Whole Ab	Cytiva	Cat# GENA934-1ML; RRID: AB_3068584
Goat Anti-Mouse IgG antibody, (H+L) FITC conjugated	Merck	Cat# AP124F; RRID: AB_92460
Mouse Anti-V5 (Clone SV5-Pk1)	Thermo Fisher Scientific	Cat# R960-25; RRID: AB_2556564
Mouse Anti-GAPDH (Clone 6C5)	Santa Cruz Biotechnology	Cat# sc-32233; RRID: AB_627679
Mouse Anti-β-tubulin (Clone D-10)	Santa Cruz Biotechnology	Cat# sc-5274; RRID: AB_2288090
Mouse Anti-GFP (Clone B-2)	Santa Cruz Biotechnology	Cat# sc-9996; RRID: AB_627695
Rabbit anti-Akt	Cell Signaling Technology	Cat# 9272; RRID: AB_329827
Rabbit Anti-Phospho-Akt S473	Cell Signaling Technology	Cat# 9271; RRID: AB_329825
Rabbit Anti-Phospho-Akt T308	Cell Signaling Technology	Cat# 9275; RRID: AB_329828
Rabbit Anti-Insulin Receptor β (Clone 4B8)	Cell Signaling Technology	Cat# 3025; RRID: AB_2280448
Rabbit Anti-Phospho-Insulin Receptor Y1361 (Clone 84B2)	Cell Signaling Technology	Cat# 3023; RRID: AB_2249189
Rabbit Anti-Phospho-Rictor Thr1135 (Clone D30A3)	Cell Signaling Technology	Cat# 3806; RRID: AB_10557237
Rabbit Anti-Phospho-Sin1 Thr86 (Clone D4U9L)	Cell Signaling Technology	Cat# 14716; RRID: AB_2798584
Mouse Anti-Rictor	Proteintech	Cat# 27248-1-AP; RRID: AB_2880817
Mouse Anti-Sin1	Proteintech	Cat# 15463-1-AP; RRID: AB_10598466
Rabbit Anti-GLUT4 (Clone 3G7C9)	Proteintech	Cat# 66846-1-lg; RRID: AB_3068586
Mouse Anti-Acetyl coA carboxylase 1	Proteintech	Cat# 21923-1-AP; RRID: AB_11042445
Mouse Anti-Stearoyl Coenzyme A Desaturase 1	Proteintech	Cat# 28678-1-AP; RRID: AB_2923581
Rabbit Anti-Mouse Albumin antibody	Bio-Rad	Cat# AHC1478; RRID: AB_3068587
**Bacterial and virus strains**		
E. coli DH5α strain	New England Biolabs	Cat# C2987H
E. coli Rosetta-2 strain	Novagen	Cat# 71397-3
AAV-CREB-H-ΔTC	Cheng. Y, et al. [25]	N/A
AAV-eGFP	Cheng. Y, et al. [25]	N/A
**Chemicals, peptides, and recombinant proteins**		
Recombinant Cgref1 Protein	This Paper	N/A
PEI Transfection Reagent	Polysciences	Cat# 23966-1
Restriction Enzyme: HindIII	New England Biolabs	Cat# R0104S
Restriction Enzyme: NotI	New England Biolabs	Cat# R0189S
Restriction Enzyme: NheI-HF	New England Biolabs	Cat# R3131S
Restriction Enzyme: XhoI	New England Biolabs	Cat# R0146S
Lipofectamine™ 2000 Transfection Reagent	Invitrogen	Cat# 11668019
LB Broth Base (Lennox)	Invitrogen	Cat# 12780052
LB Agar (Lennox L Agar), powder	Invitrogen	Cat# 22700025
Dynabeads™ M-280 Sheep Anti-Rabbit IgG	Invitrogen	Cat# 11203D
Dynabeads™ M-280 Sheep Anti-Mouse IgG	Invitrogen	Cat# 11201D

Tween-20	Invitrogen	Cat# 15514011
Glycerol	Invitrogen	Cat# 15514029
GeneJuice® Transfection Reagent	Sigma Aldrich	Cat# 70967
Ammonium Persulfate	Sigma Aldrich	Cat# A3678
Bovine Serum Albumin	Sigma Aldrich	Cat# A9418
Caesium Chloride	Sigma Aldrich	Cat# C4036
Paraformaldehyde	Sigma Aldrich	Cat# 158127
Brefeldin A	Sigma Aldrich	Cat# B6542
Insulin Solution	Sigma Aldrich	Cat# I9278
Isopropyl β-D-1-thiogalactopyranoside (IPTG)	Sigma Aldrich	Cat# I6758
D-(+)-Glucose	Sigma Aldrich	Cat# G7021
Sodium Pyruvate	Sigma-Aldrich	Cat# P8574
Sodium Deoxycholate	Sigma-Aldrich	Cat# D6750
NP-40	Sigma-Aldrich	Cat# I3021
Formalin	Sigma-Aldrich	Cat# 47068
[3H] 2-Deoxy-D-glucose	Perkin Elmer	Cat# NET549250UC
[3H] Acetic Acid, Sodium Salt	Perkin Elmer	Cat# NET003H
DMEM, powder, high glucose	Gibco	Cat# 1200046
OPTI-MEM™ Reduced Serum MEM, powder	Gibco	Cat# 22600134
Fetal Bovine Serum, qualified, standard	Gibco	Cat# 16000044
Penicillin Streptomycin	Gibco	Cat# 15140122
Immobilon®-P polyvinylidene difluoride membrane	Millipore	Cat# IPVH15150
Benzonase	Millipore	Cat# E1014
Bromophenol Blue	Millipore	Cat# ELCR00005
WesternBright ECL HRP substrate	Advansta	Cat# K-12045
RNAiso Plus	Takara	Cat# 9109
Ampicillin	MCE	HY-B0522
Kanamycin	MCE	Cat# HY-16566
Chloramphenicol	MCE	Cat# HY-B0239
Phosphate-Buffered Saline (PBS)	Formedium	Cat# PBS100L
30% Acrylamide/Bis Solution, 29:1	Bio-Rad	Cat# 1610156
TEMED	Bio-Rad	Cat# 1610801
Protein Assay Dye Reagent Concentrate	Bio-Rad	Cat# 5000006
Protease Inhibitor Cocktail (EDTA-Free, mini-Tablet)	Biotool	Cat# LP-B14011
Pierce Protease and Phosphatase Inhibitor Mini Tablets	Thermo Fisher Scientific	Cat# A32959
Pierce™ Protein G Agarose	Thermo Fisher Scientific	Cat# 20397
PageRuler™ Prestained Protein Ladder, 10 to 180 kDa	Thermo Fisher Scientific	Cat# 26616
Triton-X-100	BDH Limited	Cat# 30632
Sodium Dodecyl Sulphate	BDH Limited	Cat# 44244
Sodium Chloride	VDR Chemicals BDH	Cat# 27810.295
Monosodium Phosphate	Merck	Cat# 6345.1000
β-mercaptoethanol	Biomatik	Cat# A2008
Xylene	Duksan Chemicals	Cat# 4341
Haematoxylin	Locally Sourced	N/A
Eosin	Locally Sourced	N/A
Hydrochloric Acid	Locally Sourced	N/A
Ammonia	Locally Sourced	N/A
Glycine	Santa Cruz Biotechnology	Cat# sc-29096B
Tris Base	Santa Cruz Biotechnology	Cat# sc-3715B
EDTA	USB Corporation	Cat# 15699
Imidazole	Fisher Chemical	Cat# O3196-500

Chloroform	Supelco	Cat# 1.02445
Ethanol	Supelco	Cat# 1.00983
Methanol	RCI Labscan	Cat# AR1115
Isopropanol	RCI Labscan	Cat# AR1162
Critical commercial assays		
TB Green® Premix Ex Taq™ (Tli RNase H Plus)	Takara	Cat# RR420
PrimeScript™ RT Reagent Kit (Perfect Real Time)	Takara	Cat# RR037
The Dual-Luciferase® Reporter (DLR™) Assay System	Promega	Cat# E1910
Alexa Fluor™ 647 Protein Labeling Kit	Invitrogen	Cat# A20173
PureLink HiPure Plasmid Midiprep Kit	Invitrogen	Cat #K210004
Stanbio Cholesterol LiquiColor® Test	Stanbio	Cat# 1010-225
Stanbio Triglyceride LiquiColor® Test (Mono), 4 x 30 mL	Stanbio	Cat# 2200-430
LabAssay ™ NEFA	FUJIFILM Wako Shibayagi Corporation	Cat# 633-52001
Wide Range Mouse Insulin enzyme-linked immunosorbent assay (ELISA) Kit	ImmunoDiagnostics	Cat #32100
Deposited data		
Microarray	GEO Datasets	GEO Accession No. GSE246514
RNA-seq	GEO Datasets	GEO Accession No. GSE246511
Original Western Blot Images	Mendeley	DOI: 10.17632/2snmdx2rm7.1
Targeted GC-MS/MS	Metabolomics Workbench	datatrack_id:4421; study_id: ST002953; DOI: http://dx.doi.org/10.21228/M8X144
Untargeted MC-MS/MS	Metabolomics Workbench	datatrack_id:4418; study_id:ST002947; DOI: http://dx.doi.org/10.21228/M8X144
Experimental models: Cell lines		
Mouse: Hepa1-6	ATCC	Cat# CRL-1830; RRID: CVCL_0327
Mouse: 3T3-L1	ATCC	RRID: CVCL_0123
Human: Huh7	Locally Sourced	RRID: CVCL_0336
Human: HEK293T	ATCC	Cat# CRL-3216; RRID: CVCL_0063
Experimental models: Organisms/strains		
M. musculus: C57BL/6N	Centre of Comparative Research, HKU	N/A
M. musculus: *Creb3l3*$^{tm1.1Sad}$/J Mice	Jackson Laboratory	Strain #:010538<br>RRID: IMSR_JAX:010538
M.musculus: C57BL/6NJ-*Cgref1*$^{em1(IMPC)J}$/Mmjax	Jackson Laboratory	MMRRC Strain: #042124-JAX<br>RRID: MMRRC_042124-JAX
Oligonucleotides		
Cloning Primers (Table 1)	Integrated DNA Technologies	N/A
qPCR Primers (Table 2)	Integrated DNA Technologies	N/A
Recombinant DNA		
pET30a-His-Cgref1	Genscript	N/A
pcDNA3.1c-mCgref1-V5-His	This Paper	N/A
pcDNA3.1c-hCGREF1-V5-His	This Paper	N/A
pGL3-Cgref1-Luc-A (-130 bp pto +77 bp)	This Paper	N/A
pGL3- Cgref1-Luc-B (-384 bp to +77 bp)	This Paper	N/A
pGL3- Cgref1-Luc-C (-771 bp to +77 bp)	This Paper	N/A
pGL3- Glut4-Luc (-247 bp to -805 bp)	This Paper	N/A
pRL-SV40	Promega	Cat# E2231
pAd-RFP-Rab2	Locally Sourced	N/A
pcDNA3.1a-CREB-H-FL-V5-His	Chin, KT., et al[22]	N/A
pcDNA3.1a-CREB-H-ΔTC-V5-His	Chin, KT., et al[22]	N/A
pcDNA™3.1/V5-His A	Invitrogen	Cat# V81020
pcDNA™3.1/V5-His C	Invitrogen	Cat# V81020

Software and algorithms		
ImageJ	Rasband, W.S. [79]	https://ij.imjoy.io/
Zeiss ZEN Blue (Microscopy Software)	Centre For Panoromic Sciences, HKU	https://www.zeiss.com/microscopy/en/products/software/zeiss-zen-desk.html
Living Image 4.4 (Compatible with IVIS PE Spectrum)	Centre For Panoromic Sciences, HKU	Part No. 128113; https://www.perkinelmer.com/uk/product/spectrum-200-living-image-v4series-1-128113
Image Lab Software (Compatible with ChemiDoc MP Imaging System)	School of Biomedical Sciences, HKU	https://www.bio-rad.com/en-hk/product/image-lab-software?ID=KRE6P5E8Z
GE AKTA Purifier 10 FPLC System w/ UV-900 Detector (with Computer and Software)	School of Biomedical Sciences, HKU	https://www.marshallscientific.com/GE-AKTA-Purifier-10-FPLC-System-w-UV-900-Detecto-p/ak-p10uv.htm
Other		
X-ray Film Super RX-N	Fuji	Cat# 47410
Microseal 'B' PCR Plate Sealing Film, adhesive, optical	Bio-Rad	Cat# MSB1001
Amicon® Ultra-15 Centrifugal Filter Unit	Millipore	Cat# UFC9010
HisTrap HP His tag protein purification column	Cytiva	Cat# 17524801
3mm Stainless Steel Beads	Locally Sourced	N/A
Accu-Chek Guide Glucose Meter	Roche	N/A
Accu-Chek Guide Test Strips	Roche	N/A

Table S2. Cloning primers used in this study

Plasmid	Forward	Reverse
pcDNA3.1c-mCgref1-V5-His	5′-TATCTAAGCTTATGTTCCAGTGGCTGATGC-3′	5′-TATCTGCGGCCGCTATCTCATCGTTCTCCAATTGGATG-3′
pcDNA3.1c-hCGREF1-V5-His	5′- TATCTAAGCTTATGTTACCTTTGACGATGACAGTG-3′	5′-TATCTGCGGCCGCGATCTCATCATTCTCCACTTGAACAAT-3′
pGL3-Cgref1-Luc-A (-130 bp to +77 bp)	5′-TATCTGCTAGCGAACAAGGCCAGGTGGAGG-3′	5′-TATCTAAGCTTTGAACTCTATAGGCACCGCG-3′
pGL3- Cgref1-Luc-B (-384 bp to +77 bp)	5′-TATCTGCTAGCACATGCACATCAGACTCACCA-3′	
pGL3- Cgref1-Luc-C (-771 bp to +77 bp)	5′-TATCTGCTAGCCCCGTACATGAAGCCTCCTC-3′	
pGL3- Glut4-Luc-C	5′-TATCTCTCGAGTGCGTGGAAAGAAAGGACGA-3′	5′-TATCTAAGCTTTAGGACACGCCCTTCACAAC-3′

Table S3. qPCR primers used in this study

Target	Forward Primer	Reverse Primer
Cgref1 (mouse)	5′-GCTCCAGTTTCCAGCAGGAT-3′	5′-TGGAAAGGGTTGGGTGTGAG-3′
CGREF1 (human)	5′-CAAAGGATGGAGTCACAAGGC-3′	5′-GAAGGGGTTGGGCAGGAG-3′
Scd1	5′-GCCCACATGCTCCAAGAGAT-3′	5′-CTTTGACAGCCGGGTGTTTG-3′
Acc1	5′-TTGCCATGGGGATCCCTCTA-3′	5′-GCTGTTCCTCAGGCTCACAT-3′
Acc2	5′-CCTGAATGTGGCCATCCAGT-3′	5′-GACTCCTCGATCTTGAGCGG-3′
Fasn	5′-GGCCCCTCTGTTAATTGGCT-3′	5′-CGCTTGTTGGTGGACACTTG-3′
Acly	5′-TCCCCATCCATGTCTTTGGC-3′	5′-GATCAGCACGTCTACCTCCG-3′
Elovl6	5′-ATCTTTGGTGGTCGGCATCT-3′	5′-ACAGCCCATCAGCATCTGAG-3′
Glut4	5′-AACACTCAACCAACTGGCCA-3′	5′-CACCGAGACCAACGTGAAGA-3′
Srebp	5′-GACCCTACGAAGTGCACACA-3′	5′-GTGGCCTAGTCACAGGTTCC-3′
Chrebp	5′-CCCGTCCCCTTCTCTGTAGA-3′	5′-TTGTTGTCTACACGACCCCG-3′
Gapdh	5′-TTCACCACCATGGAGAAGGC-3′	5′-TGAAGTCGCAGGAGACAACC-3′
Hprt	5′-CAGTCCCAGCGTCGTGATTA-3′	5′-TCCAACAAAGTCTGGCCTGT-3′
hAAT	5′-TTTCGGTAAGTGCAGTGGAAG-3′	5′-TTATCGGAGGAGCAAACAGG-3′
CREB-H (WT genotyping)	5′-TGAACCAGCCAACACGGTAG-3′	5′-TTGTCCTTCTCGGGGCAGA-3′
CREB-H (KO genotyping)	5′-AAGTAATCCCCTGCCTCCAC-3′	5′-GCTGGGAAGACTGAGACTGG-3′
Cgref1 (WT genotyping)	5′-TGAGCAGGCATCTTTCCTCC-3′	5′-TCACACTGGGCTAGAGCTGA-3′
Cgref1 (KO genotyping)	5′-CTCTGTTGAGGGGCATCTGG-3′	5′-GTTTGGTCACTGTTGCCAGC-3′
mCgref1-ChIP (promoter -532 to -451)	5′-TCTCAGATAAAGCCAGTGACAC-3′	5′-AAAACTAGACAGATGTGAGGCT-3′
mCgref1-ChIP (promter -272 to -156)	5′-AAATCCTTCCCAGCCTGTCA-3′	5′-GGAGTCCTCAGTGTGTGTCA-3′
mCgref1-ChIP (CDS)	5′-GACTCTCCCTGCTGACTTCC-3′	5′-TCCCTTCCTGTATCCTCCCA-3′

SUPPLEMNETARY FIGURES

A

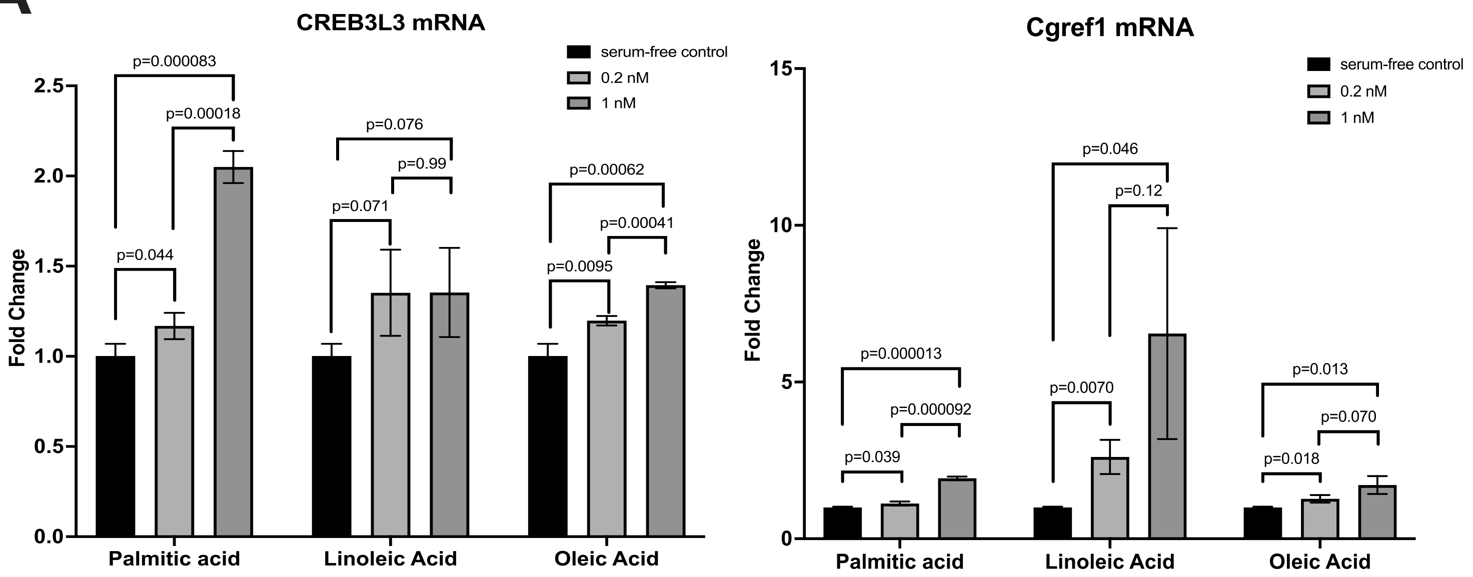

B

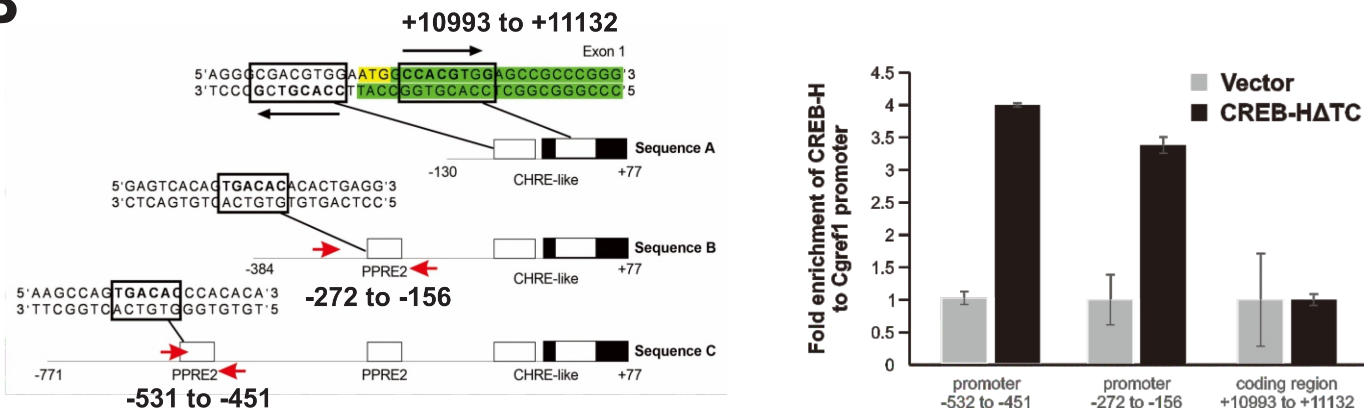

Figure S1. Cgref1 expression is transcriptionally regulated by CREB-H. (A) Cgref1 and CREB-H mRNA expression in fatty acid-treated Hep3B cells. The culture medium was replaced with serum-free DMEM containing 1% fatty acid-free BSA 30 minutes before treatment. The Hep3B cells were then exposed to escalating concentrations (0.2 nM and 1 nM) of fatty acids (palmitic acid, linoleic acid, and oleic acid) or mock-treated for an additional 6 hours. After treatment, the cells were harvested for RNA extraction and RT-qPCR analysis. (B) Recruitment of CREB-H to the *Cgref1* gene promoter. Hepa1-6 cells were transfected with either empty vector or CREB-HΔTC-V5 expression plasmid. After 40 hours, cells were crosslinked with formaldehyde to stabilize DNA-protein interactions. Chromatin was extracted, sonicated, and incubated with V5 antibody-bound magnetic beads to pull down CREB-H-associated DNA. DNA was purified and analyzed by RT-qPCR using primers targeting two sites in the Cgref1 promoter and one site in the coding region for normalization. Results were normalized against the empty vector control to account for non-specific binding. This assay demonstrated the specific binding of CREB-H to the promoter regions of the *Cgref1* gene.

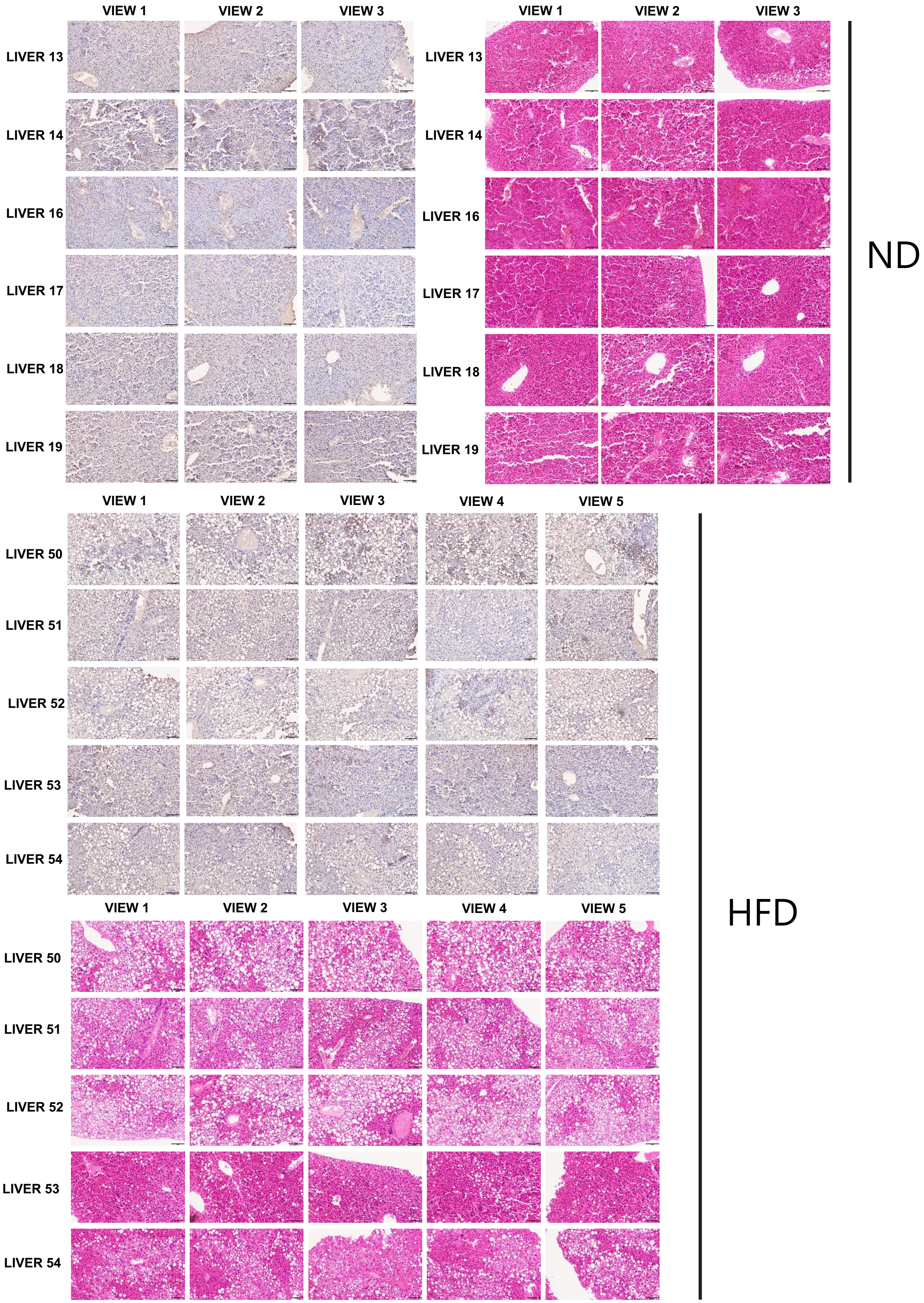

Figure S2. Cgref1 expression is enhanced by HFD consumption. Liver tissues from mice given ND and HFD were analyzed and compared by immunohistochemical and H&E staining.

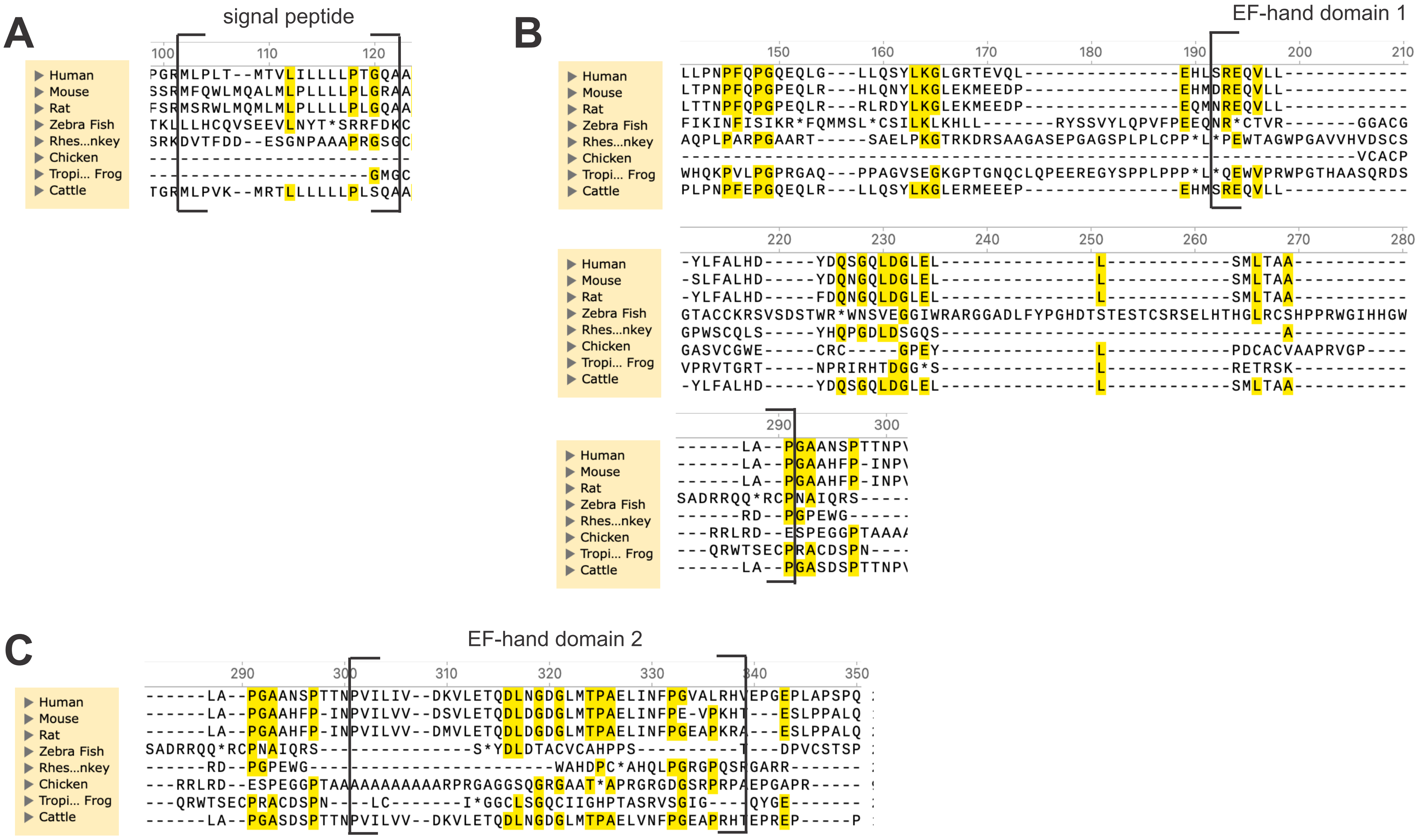

Figure S3. The functional domains of Cgref1. (A-C) Multiple sequence alignments of the signal peptide (SP) (A), EF-hand domain 1 (B) and EF-hand domain 2 (C) of Cgref1 across different living species.

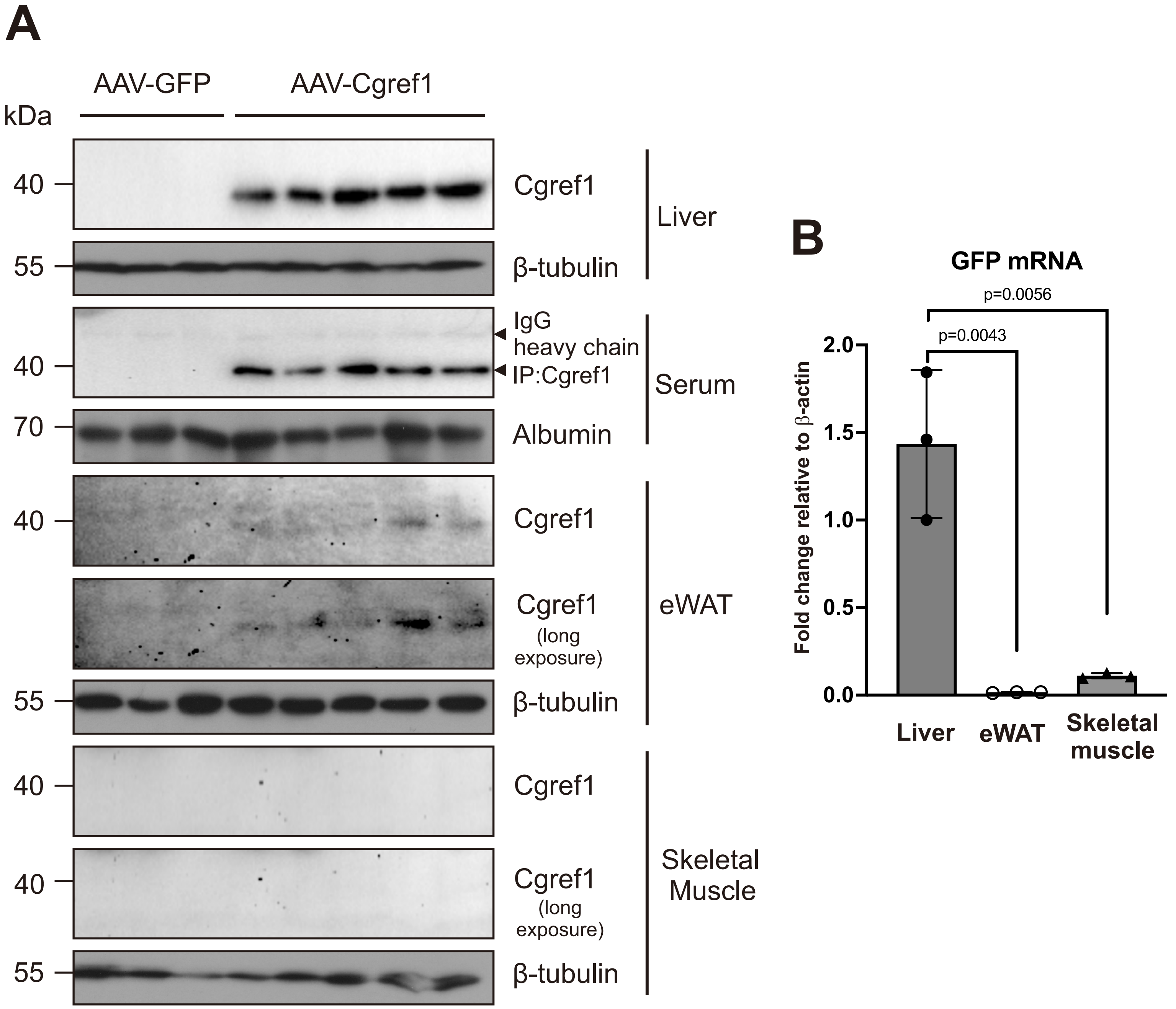

Figure S4. Liver-produced Cgref1 is secreted into the circulation and reaches the eWAT. (A) *Cgref1*$^{-/-}$ mice received intraperitoneal injection of 1×10^{11} genome copies of liver-targeting AAV-GFP or AAV-Cgref1. Two weeks later, liver, eWAT, skeletal muscle tissues and sera were collected for Western blot analysis. (B) Liver-specific expression of Cgref1. The GFP marker was also expressed from the AAV vector. RT-qPCR analysis confirmed that GFP was expressed in the liver, but not eWAT or skeletal muscle.

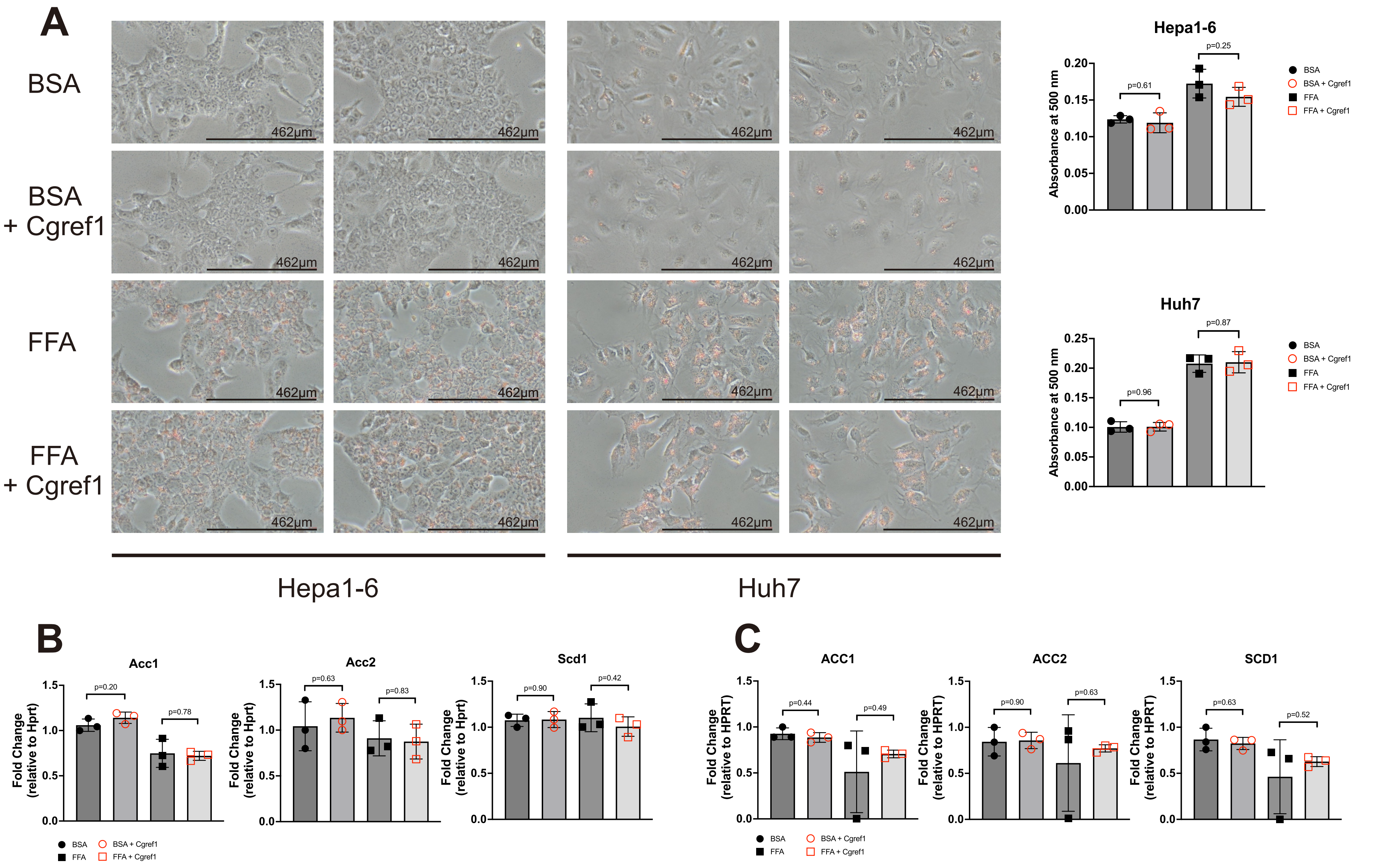

Figure S5. Cgref1 has no direct effects on hepatic lipogenesis. Recombinant Cgref1 protein treatment (10 μg/ml) of Hepa1-6 and Huh7 hepatoma cell lines in either 1% fatty acid-free BSA or 1% fatty acid-free BSA supplemented with 0.5 mM oleic acid and 0.25 mM palmitic acid for 24 hours. Lipogenesis was compared by (A) spectrophotometry of oil red O staining, and (B and C) RT-qPCR of mouse and human homologs of *de novo* lipogenesis genes expressed in both cell lines.

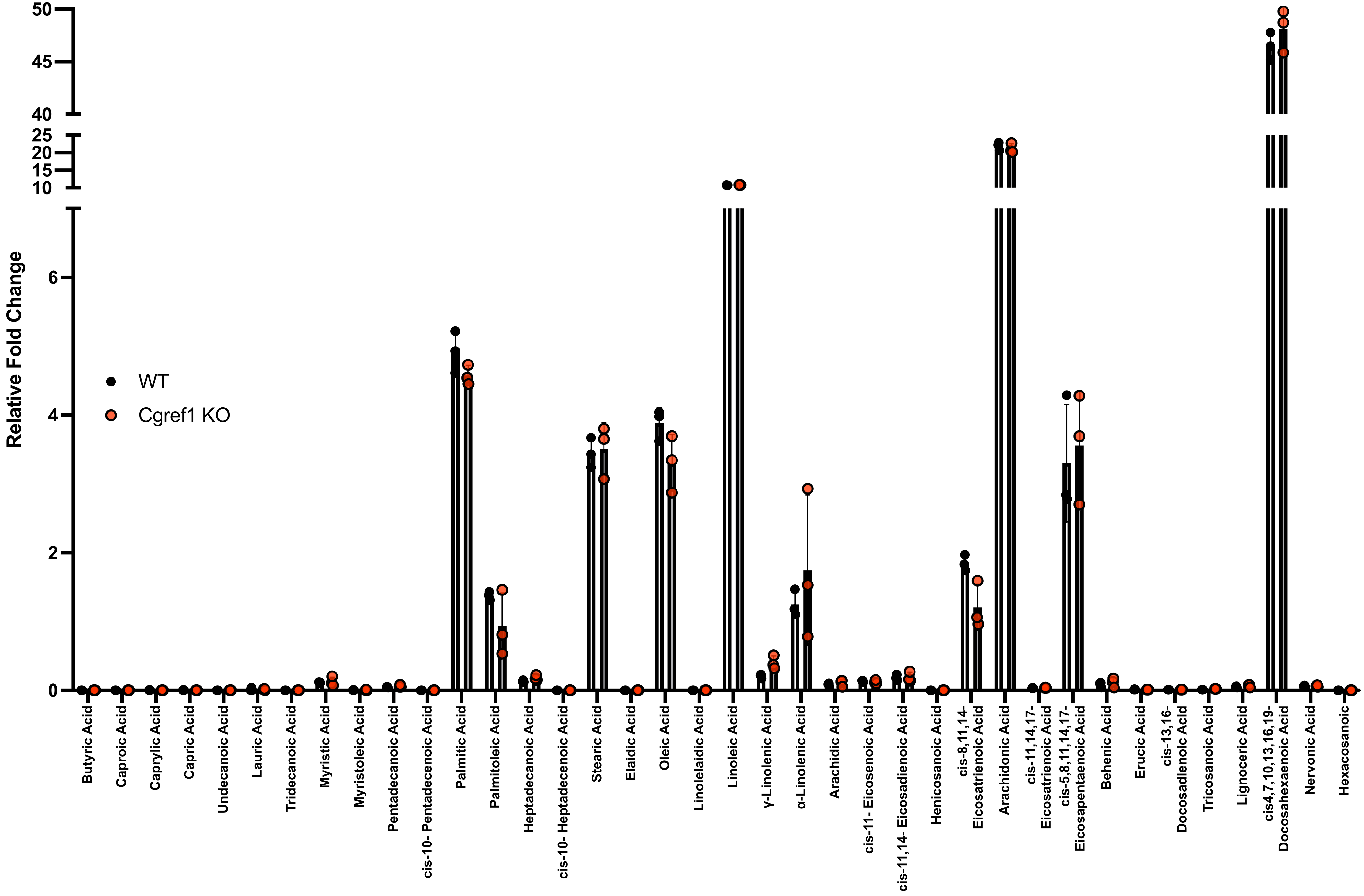

Figure S6. Analysis of hepatic medium or long chain fatty acid profile. Gas chromatography mass spectrometry (GC-MS/MS) analysis of hepatic medium or long chain fatty acids in WT and *Cgref1*$^{-/-}$ mice (n = 3 per group).

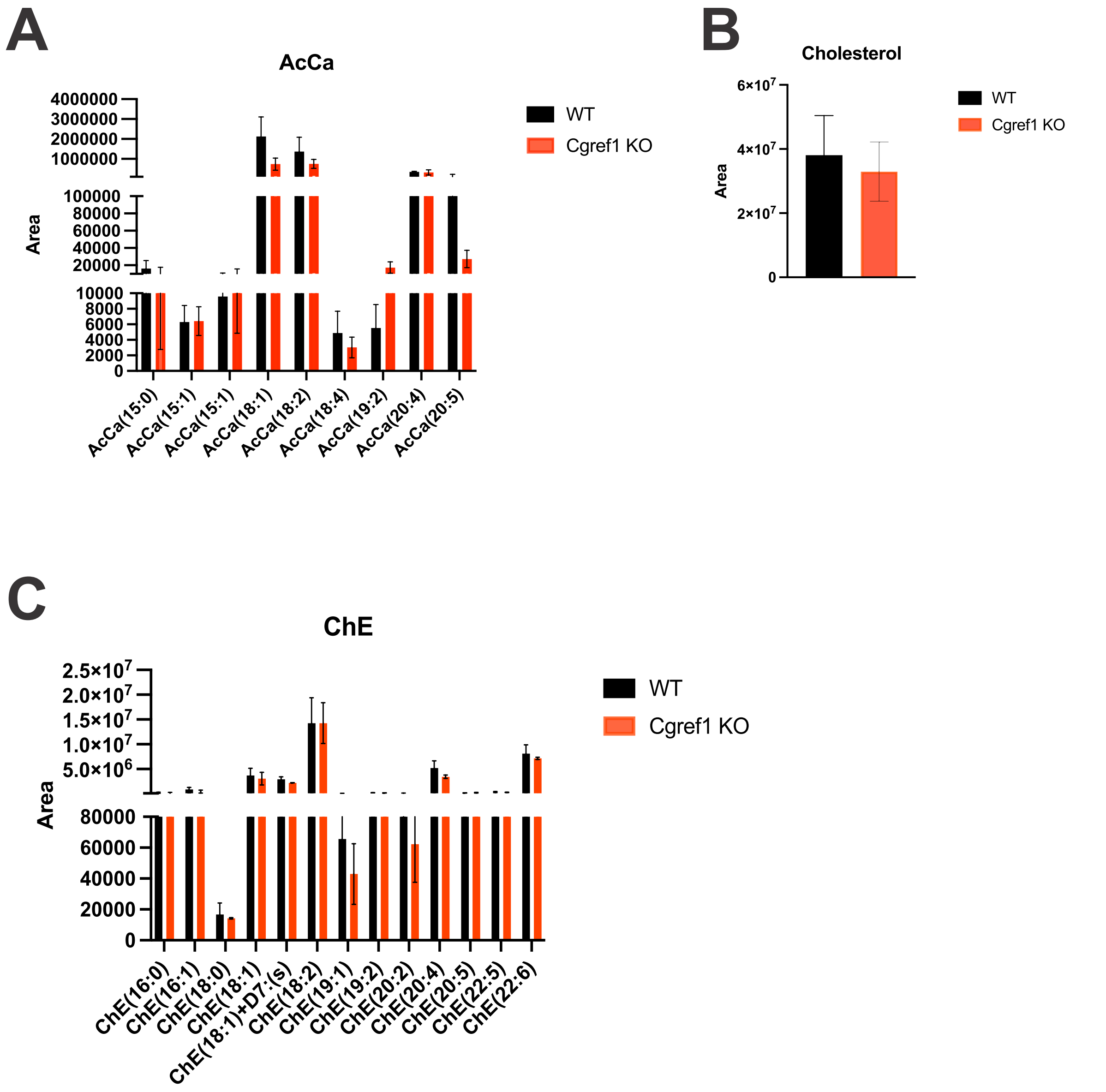

Figure S7. Cytosolic lipid profile. (A-C) LC-MS/MS analyses of acylcarnitines (AcCa) (A), cholesteryl esters (CE) (B) and cholesterol (ChE) (C) in the livers of WT and *Cgref1*$^{-/-}$ mice (n = 3 per group).

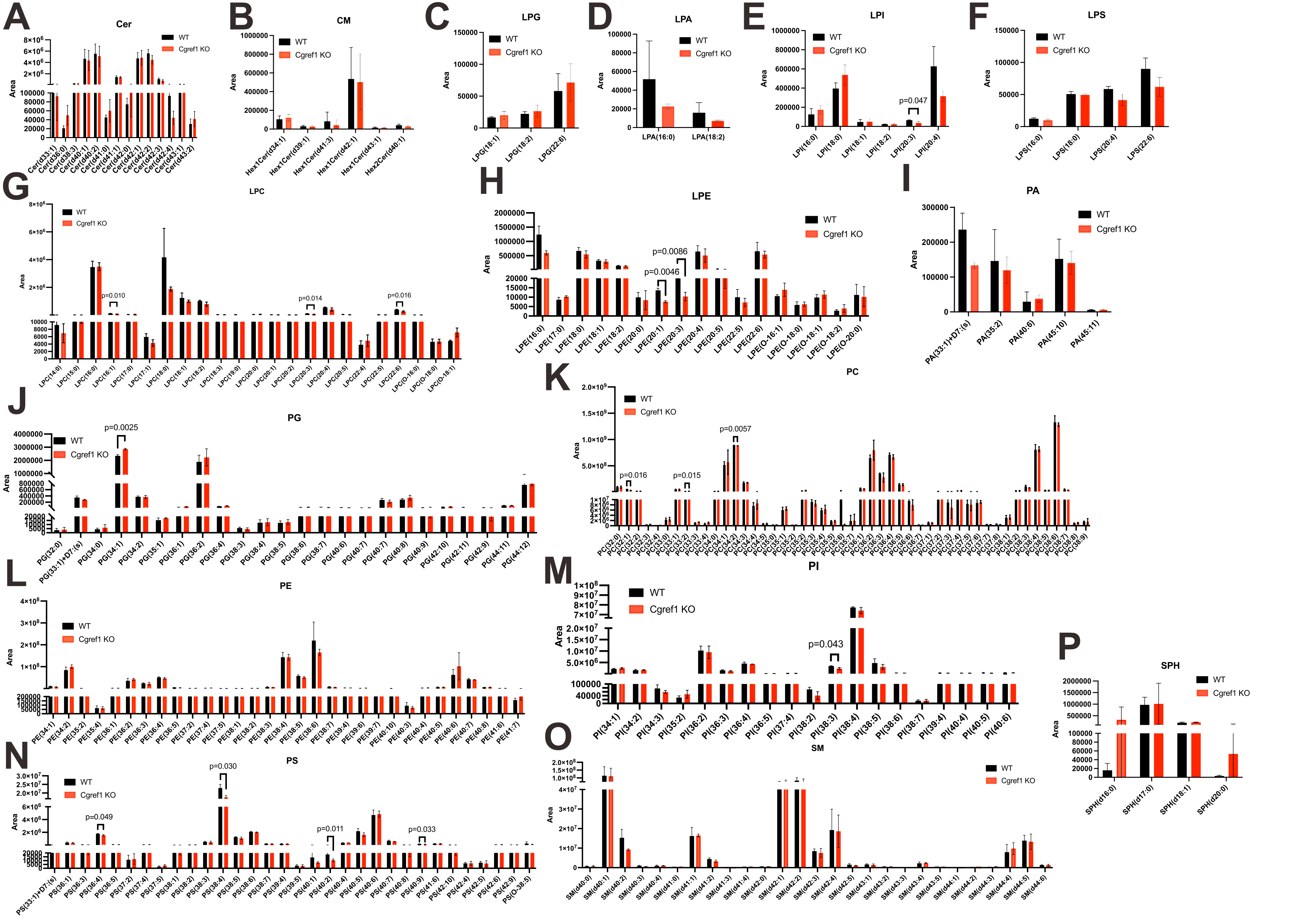

Figure S8. Membrane lipid profile. (A-P) LC-MS/MS analyses of ceramide (Cer) (A), ceramide monosaccharides (CM) (B), lysyl-phosphatidylglycerol (LPG) (C), lysophosphatidic acid (LPA) (D), lysophosphatidyl-inositols (LPI) (E), lipopolysaccharide (LPS) (F), lysophosphatidylcholine (LPC) (G), lysophosphatidylethanolamine (LPE) (H), phosphatidic acid (PA) (I), phosphatidylglycerol (PG) (J), phosphatidylcholine (PC) (K), phosphatidylethanolamine (PE) (L), phosphoinositides (PI) (M), phosphatidylserine (PS) (N), sphingomyelin (SM) (O) and sphingosine (SPH) (P) in livers of WT and *Cgref1*$^{-/-}$ mice.
